# The anterior chamber of the eye technology and its anatomical, optical, and immunological bases

**DOI:** 10.1152/physrev.00024.2023

**Published:** 2024-01-11

**Authors:** Shao-Nian Yang, Yue Shi, Per-Olof Berggren

**Affiliations:** The Rolf Luft Research Center for Diabetes and Endocrinology, Karolinska Institutet, Stockholm, Sweden

**Keywords:** in vivo microimaging, innervation, the anterior chamber of the eye, transplantation, vascularization

## Abstract

The anterior chamber of the eye (ACE) is distinct in its anatomy, optics, and immunology. This guarantees that the eye perceives visual information in the context of physiology even when encountering adverse incidents like inflammation. In addition, this endows the ACE with the special nursery bed iris enriched in vasculatures and nerves. The ACE constitutes a confined space enclosing an oxygen/nutrient-rich, immune-privileged, and less stressful milieu as well as an optically transparent medium. Therefore, aside from visual perception, the ACE unexpectedly serves as an excellent transplantation site for different body parts and a unique platform for noninvasive, longitudinal, and intravital microimaging of different grafts. On the basis of these merits, the ACE technology has evolved from the prototypical through the conventional to the advanced version. Studies using this technology as a versatile biomedical research platform have led to a diverse range of basic knowledge and in-depth understanding of a variety of cells, tissues, and organs as well as artificial biomaterials, pharmaceuticals, and abiotic substances. Remarkably, the technology turns in vivo dynamic imaging of the morphological characteristics, organotypic features, developmental fates, and specific functions of intracameral grafts into reality under physiological and pathological conditions. Here we review the anatomical, optical, and immunological bases as well as technical details of the ACE technology. Moreover, we discuss major achievements obtained and potential prospective avenues for this technology.

## Graphical abstract

**Figure:**
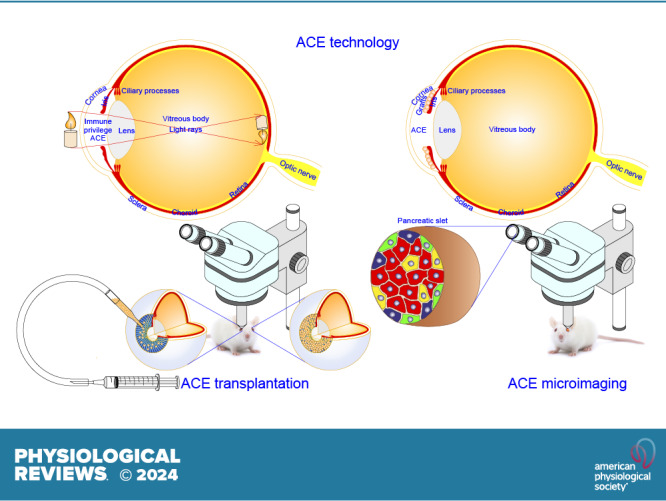

CLINICAL HIGHLIGHTS

The anterior chamber of the eye (ACE) technology has since long been used to study various cells, tissues, and organs as well as artificial biomaterials, pharmaceuticals, and abiotic substances not only under physiological but also under pathological conditions. It is feasible to perform in vivo studies on human cells, tissues, and organs engrafted in the immunodeficient mouse ACE without violating ethical standards. The technology is versatile in biomedical research on intracameral grafts from different parts of the human body. It can be used to model human diseases with human cells, tissues, and organs engrafted in immunodeficient mouse recipients. Clinical trials of human islet transplantation into the ACE of patients with diabetes are ongoing and their outcomes are expected to be superior to those into the conventional site, namely the hepatic portal system of the recipient patient.

## 1. INTRODUCTION

**FIGURE 1.**
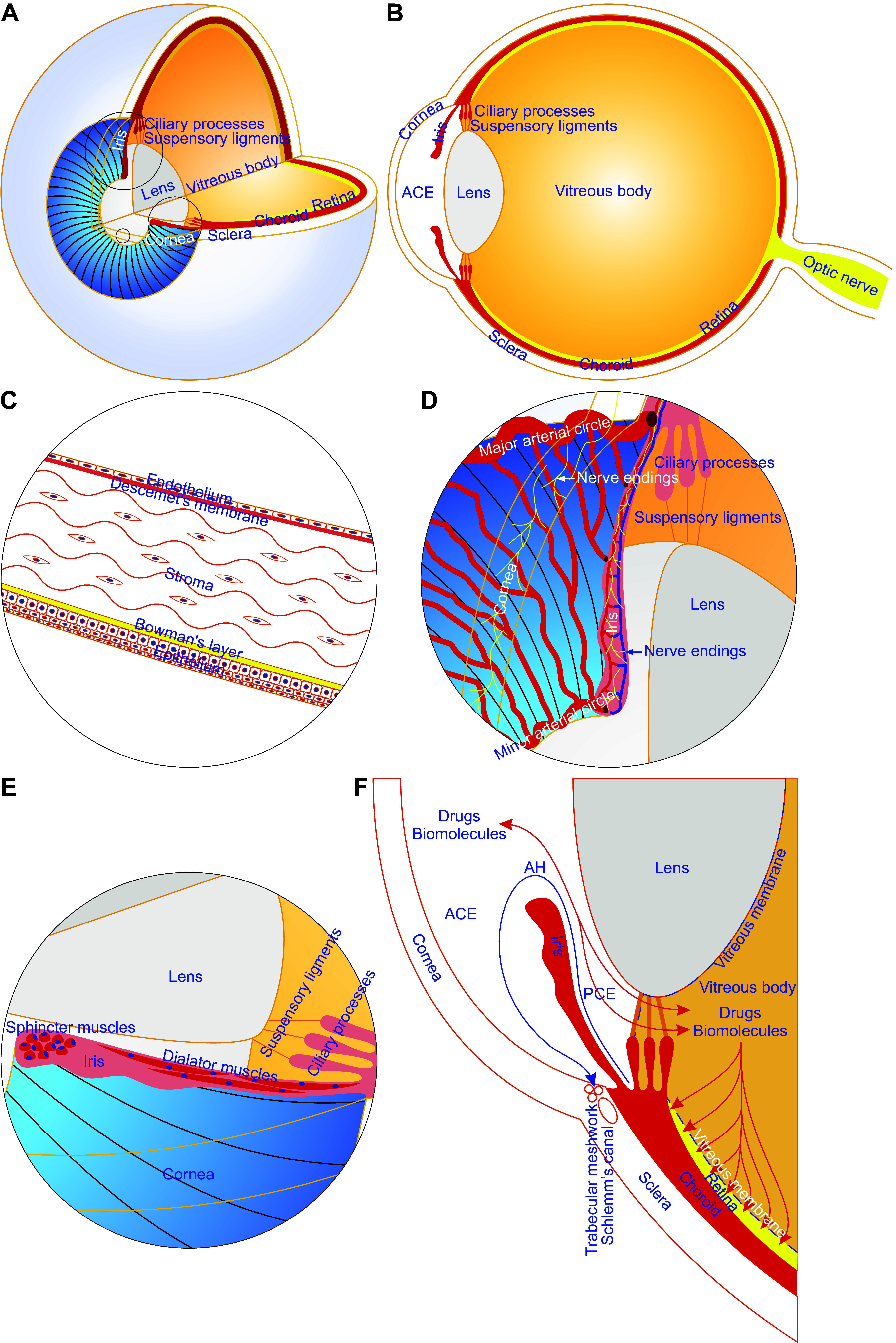
Anatomical features of the anterior chamber of the eye (ACE). The ACE is built up mainly of the cornea, iris, and aqueous humor (AH). The cornea is avascular and consists of a thin nonkeratinized epithelium, acellular Bowman’s layer, stroma with highly organized collagen fibrils and very few keratocytes, cell-free matrix-formed Descemet’s membrane, and endothelial cell monolayer. Hence, the cornea is characterized as a transparent natural body window that allows light to reach the retina and gives rise to a key prerequisite for noninvasive, longitudinal, and intravital microimaging of intracameral grafts. The iris is richly vascularized and innervated. It can provide a major engraftment area for intracameral grafts and predominately contributes to intracameral graft vascularization and innervation, thereby acting as a favorable graft survival substratum. The AH is a water-like liquid, produced by the ciliary processes, flows into the posterior chamber of the eye and then the ACE through the pupil and eventually drains sequentially into trabecular meshworks, Schlemm’s canal, and episcleral veins. The AH supplies oxygen, nutrients, and other survival factors to and removes metabolic wastes from intracameral grafts. Of note, the vitreous membrane is porous and allows diffusion of intraocular drugs or biomolecules between the anterior/posterior chambers, vitreous body, and retinal compartment. *A* and *B:* three-fourths 3-dimensional and one-half sagittal views of the eyeball showing the structure of the ACE. *C:* partially enlarged view of the small circle area in *A* showing the 5-layer architecture of the cornea. *D* and *E:* partially enlarged view of the large and middle circle areas in *A* showing iridic vasculatures, nerve endings, and smooth muscles. *F:* sectional view of the partial lower half eyeball illustrating the drainage path for AH and the diffusion paths of intraocular drugs and biomolecules between intraocular compartments. PCE, posterior chamber of the eye.

**FIGURE 2.**
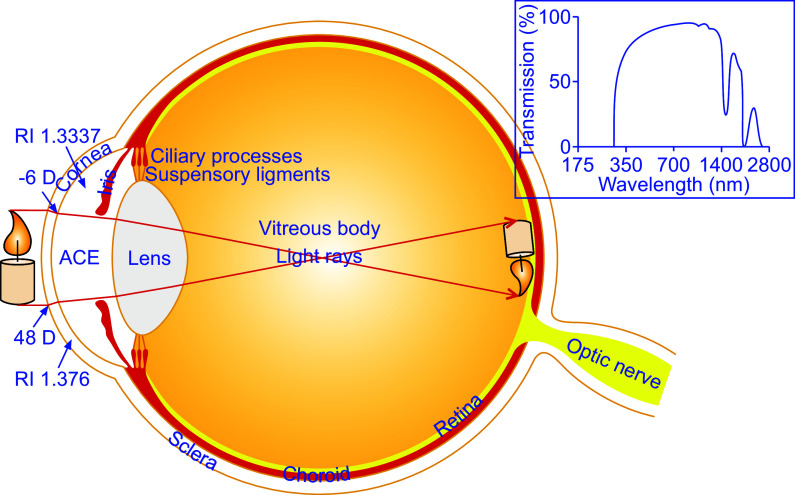
The optical quality of the anterior chamber of the eye (ACE). The transparency and absorbance of the cornea and aqueous humor (AH) as well as the shape of the former determine the optical quality of the ACE. The cornea refracts light twice when entering and leaving the cornea. Light enters the cornea at the greater angle than it leaves it because the difference in refractive index (RI) between cornea and AH is smaller than that between the air and cornea. The cornea and AH also serve as filters rejecting some of the most damaging ultraviolet rays and getting rid of significant amounts of infrared waves at 1,430 nm and 1,950 nm. D, diopter. *Inset* is adapted from Ref. 66, with permission.

**FIGURE 3.**
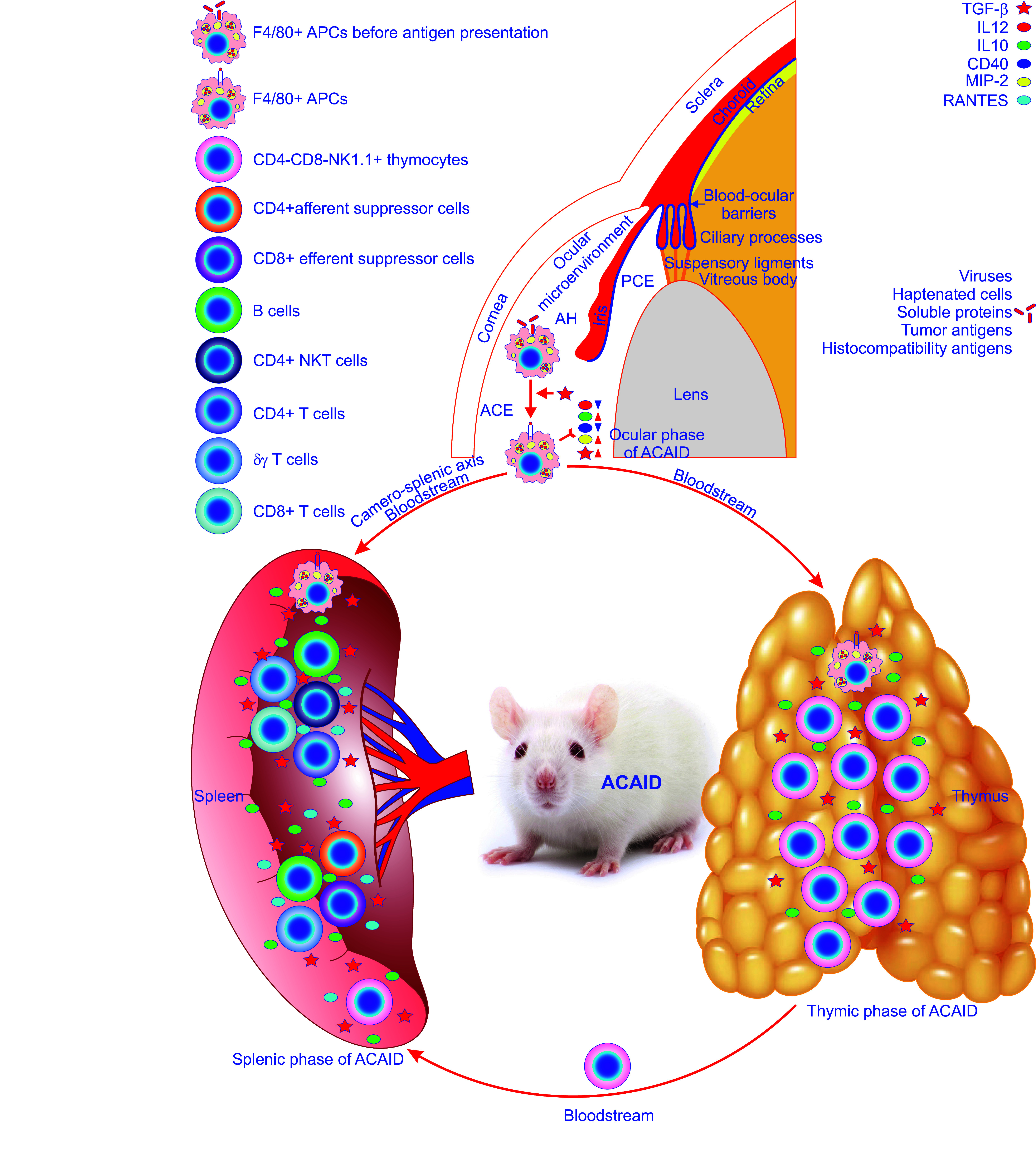
The constitution of ocular immune privilege. Ocular immune privilege is attributed to a diverse range of ocular and systemic mechanisms, 3 of which stand out. The first one sequestrates antigens entering the eye due to efficient blood-ocular barriers, including the blood-retinal barrier and the blood-aqueous barrier, and the lack of efferent lymphatics. These 2 barriers are emphasized by a blue line in the eyeball. The second one manifests as active immune suppression by antigens, including viruses, haptenated cells, soluble proteins, tumor antigens, and histocompatibility antigens, invading the anterior chamber of the eye (ACE), i.e., the anterior chamber-associated immune deviation (ACAID). ACAID can be divided into the ocular, thymic, and splenic phases. It starts from the ocular phase where F4/80+ ocular antigen-presenting cells (APCs) capture, process and present invading antigens thereby being primed under regulation of various cytokines and chemokines such as transforming growth factor-β (TGF-β). These primed ocular APCs release less T_H_1-inducing cytokine IL-12 and CD40 costimulatory molecule and more cytokine IL-10 and become able to produce TGF-β and macrophage inflammatory protein-2 (MIP-2; *top*). Then, they escape from the ACE into the thymus and spleen through the bloodstream. The latter route is termed the camero-splenic axis. Upon entry into the thymus, the primed ocular APCs induce the thymic phase of ACAID resulting in the generation of CD4-CD8-NK1.1+ thymocytes. Subsequently, these thymocytes flow into the bloodstream and home to the spleen to participate in the splenic phase of ACAID. As soon as the primed F4/80+ ocular APCs reach the spleen, the splenic phase of ACAID commences. In this important phase, F4/80+ APCs, CD4-CD8-NK1.1+ thymocytes, B cells, CD4+ natural killer (NK)T cells, CD4+ T cells, δγ T cells, and CD8+ T cells interact with each other in a milieu enriched in TGF-β, IL12, IL10, CD40, MIP-2, and regulated on activation normal T-expressed and presumably secreted (RANTES). Eventually, this complex immune cell interaction reaches a finale with the production of CD4+ afferent and CD8+ efferent suppressor cells, the former suppressing the induction of DTH responses whereas the latter inhibiting the expression of DTH responses, thereby ultimately resulting in ACAID. The third one is the ocular microenvironment created by ocular cells, such as corneal endothelial cells, iris pigment epithelial cells, ciliary body pigment epithelial cells, and retinal pigment epithelial cells, which secrete immunosuppressive factors and mediate contact-dependent immune suppression. AH, aqueous humor.

The anterior chamber of the eye (ACE) serves as the entrance for visual information to the brain (FIGURES 1 AND 2) (1, 2). Beyond that, the ACE has been found to be a unique transplantation site due to its anatomical and immunological features (FIGURES 1 AND 3) (3). Most obviously, the ACE is specifically fitted with a nutrient/oxygen-rich milieu and an immune-privileged niche where appropriately sized grafts like pancreatic islets, small chunks of brain and tumor tissues, as well as engineered organoids can survive reasonably well before graft vascularization (4–13). Furthermore, the iris can be used as a favorable substrate to support permanent engraftment and a rich source of blood vessels and nerves for intracameral graft vascularization and innervation, thereby enabling the prolonged survival of functional grafts (4–6).

Increasing evidence shows that cells, tissues, and organs can behave differently in vitro versus in vivo (6, 14). However, noninvasively and longitudinally microimaging the structure and function of cells integrated into the living body has been a long-standing challenge. This is at least in part due to the lack of appropriate intravital microimaging platforms and the presence of insurmountable anatomical obstacles (6, 14). These issues have been solved by taking advantage of the unique optical quality of the ACE in combination with microscopy (4–6). Confocal and multiphoton fluorescence microscopy can work with their full power to noninvasively acquire high-quality and high-resolution three-dimensional (3-D) images of various grafts placed into the ACE in a longitudinal manner (4–6, 15).

Thus far, a wide range of knowledge on different physiological processes and pathological changes in vivo has been acquired by grafting different cells, tissues, and organs into the ACE. A series of seminal discoveries have emerged as a consequence of these studies (4–13, 15–37). One of the most striking examples is the development of the concept of ocular immune privilege (3, 38–57). In recent years, the ACE has become an in vivo versatile platform for different biomedical studies and in particular microscopic investigations of cells as well as pieces of tissues and organs from different body parts as well as artificial biomaterials and pharmaceuticals. This has led to the understanding of physiological events, pathological processes, disease modeling, mechanisms, diagnosis, and therapies at cellular and even subcellular levels in the in vivo context (4–7, 15–37). Importantly, it has also satisfactorily been used in in vivo studies of nonhuman primates (27, 28). Without violating ethical standards, human cells, tissues, and organs have been transplanted into the ACE of immune-compromised mice (26, 58). Such studies have laid the foundation for its clinical application for treating diabetes and other diseases (26–28, 58). It is the right time to coin the simple but meaningful term “ACE technology” (4–7, 21, 22, 34, 36, 37, 59–63).

This review aims to summarize our knowledge of the ACE technology and its anatomical, optical, and immunological bases.

## 2. ANATOMICAL AND OPTICAL FEATURES OF THE ACE

The ACE is formed by the cornea and the iris together with the lens, ciliary body, and the anterior portion of the sclera and filled with aqueous humor (AH) to transmit light from external objects to the retina (FIGURES 1 AND 2) (1). In addition, the ACE also bears a range of intrinsic merits for transplantation and in vivo microimaging (6). The anatomical benefits of using the ACE as a transplantation site and the optical rationale for employing the ACE as a novel microimaging platform are attributed to the intrinsic characteristics of individual components of the ACE, the most important of which are discussed below.

### 2.1. Cornea

The cornea is transparent, prolate in shape, and somewhat thinner at the center than in the periphery and serves as the “front window” of the ACE (FIGURES 1 AND 2) (64–67). The human cornea consists of five layers (FIGURE 1*C*) (64–67). The corneal epithelium forms the outermost layer comprising nonkeratinized epithelial cells. They are interconnected by tight junctions constituting an exceedingly thin coating on the cornea acting as an effective barrier against fluid loss from and pathogen invasion into the eye (64–67). The innermost layer of the cornea, referred to as the corneal endothelium, is composed of a simple squamous or low cuboidal monolayer of endothelial cells that are critical for regulating fluid and solute transport from the corneal stroma to the AH to maintain stromal transparency by keeping the stroma relatively dehydrated (64–67). Between the outermost and innermost layers, there are Bowman’s layer, the corneal stroma, and Descemet’s membrane. These three layers mainly consist of collagen fibrils (FIGURE 1*C*) (64–67).

**FIGURE 4.**
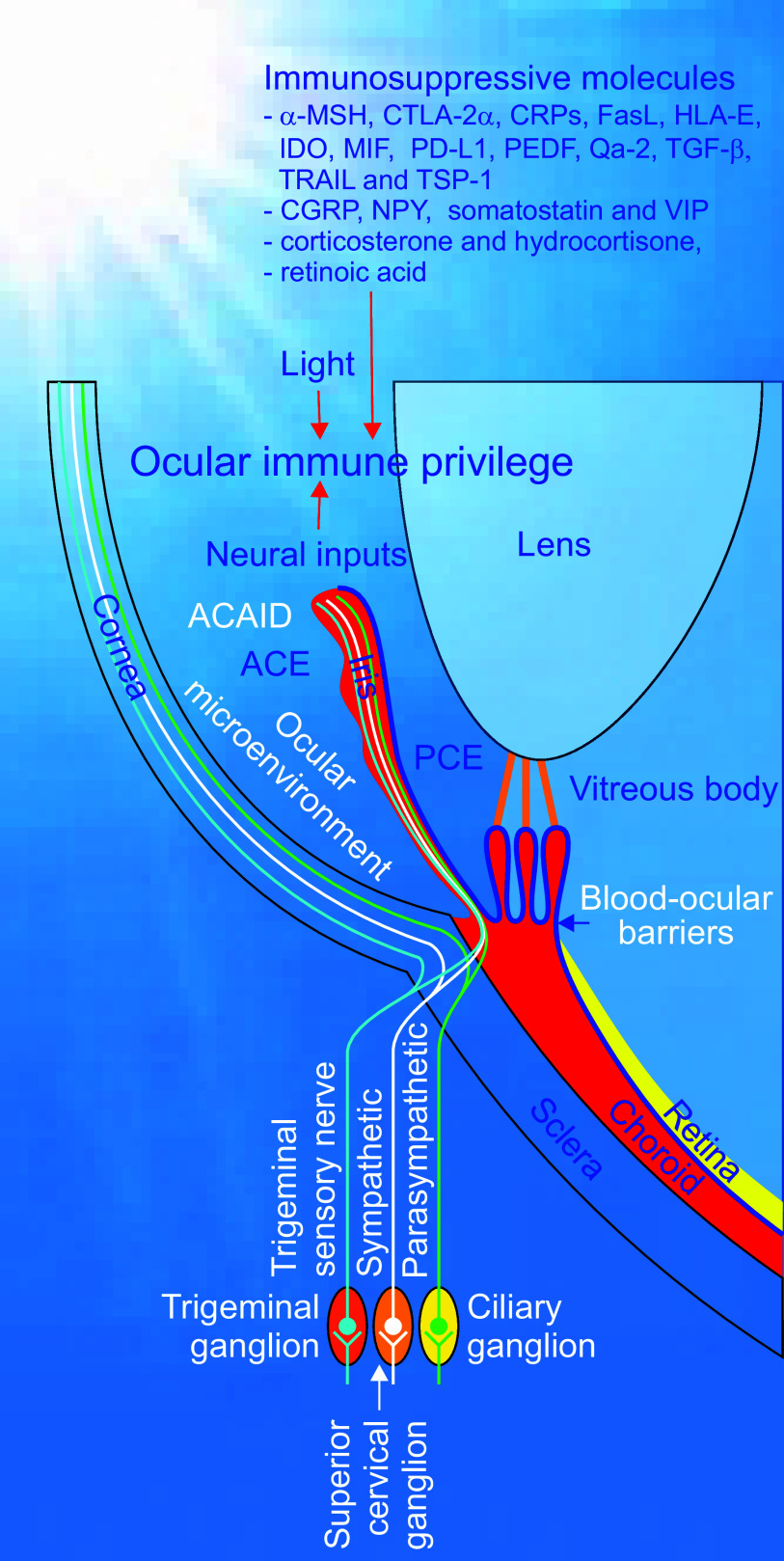
The regulation of ocular immune privilege. Ocular immune privilege is tightly regulated by immunosuppressive molecules including cytokines, chemokines, lymphokines, neuropeptides, or other anti-inflammatory species as well as by various signals derived from light exposure and neural inputs from sympathetic, parasympathetic, and sensory nerves to maintain the ocular immunological homeostasis. ACE, anterior chamber of the eye; PCE, posterior chamber of the eye; α-MSH, α-melanocyte stimulating hormone; CGRP, calcitonin gene-related protein; CTLA-2α, cytotoxic T lymphocyte antigen-2α; CRPs, complement regulatory proteins; FasL, Fas ligand; HLA-E, human leukocyte antigen E; IDO, indoleamine dioxygenase; MIF, macrophage migration inhibitory factor; NPY, neuropeptide Y; PD-L1, programmed death-ligand 1; PEDF, pigment epithelium-derived factor; TGF-β, transforming growth factor-β; TRAIL, tumor necrosis factor-related apoptosis-inducing ligand; TSP-1, thrombospondin-1; VIP, vasoactive intestinal peptide.

The cornea is one of the few avascular tissues of the body (FIGURE 1) (64, 65). It functions and survives well without blood and lymphatic vessels. This avascular tissue gets nutrients from the tear fluid and AH (68, 69). However, as the most densely innervated tissue in the body, the cornea possesses the richest sensory nerve terminals, extended from the ophthalmic division of the trigeminal nerve, and some sympathetic and parasympathetic nerve endings from the trigeminal ganglion, the superior cervical ganglion, and the ciliary ganglion, respectively (FIGURE 4). They protect the cornea by detecting and escaping from noxious stimuli and release neuropeptides and neurotransmitters, yet their potential roles remain poorly understood (64, 70). In addition, the cornea also resists immune rejection due to its intrinsic immunological properties (69).

The cornea is not only strong and durable but also as smooth and clear as glass. It serves as the eye’s outermost lens, which controls and focuses the entry of light into the eye and contributes to about two-thirds of the eye’s total focusing power (FIGURE 2). Light is refracted twice toward the midline when it enters the cornea and again when it leaves the cornea. Light enters the cornea at the greatest angle. The refractive index (RI) of the cornea is 1.376, whereas that of the AH is 1.334 (FIGURE 2) (71). The difference in RI between the cornea and AH is relatively small in comparison to that between the air and cornea. The former only brings about a negligible refractive effect, typically −6 diopters (D), whereas the latter produces a significant one (48 D) (FIGURE 2). Therefore, the cornea acts as a positive meniscus lens (FIGURE 2) (64–67, 71, 72). The cornea also acts as a filter, getting rid of some of the most damaging ultraviolet (UV) wavelengths in sunlight (FIGURE 2). In the absence of such a filter, the lens and the retina would be highly vulnerable to UV radiation (66, 73, 74). Studies on the transmission of the human cornea for light in the wavelength range of 220–2,800 nm show that UVA (315–400 nm) can pass through the cornea, but very little UVB (280–315 nm) and UVC (200–280 nm) can do so (FIGURE 2). This is because the epithelium and stroma contain some proteins and vitamins, which can effectively absorb UVB and UVC for the protection of the eye. In fact, the corneal transmittance undergoes a rapid increase from 300 nm and reaches ∼80% at 380 nm and greater than 90% between 500 nm and 1,300 nm. However, two sudden nadirs in infrared transmitted intensity occur at 1,430 nm and 1,950 nm most likely due to water absorption (FIGURE 2) (66, 73, 74). Especially noteworthy is that this phenomenon can significantly interfere with microimaging and photo manipulation of intracameral grafts illuminated by light at the wavelength 1,430 nm or 1,950 nm.

The aforementioned anatomical and optical features guarantee corneal transparency, which is of prime importance for visual acuity. As a bonus, they make optical monitoring/manipulation of grafts in the ACE possible (6).

### 2.2. Iris

The iris is a contractile muscular diaphragm with a circular central opening pupil like a “doorway” for light and AH in front of the lens and forms the posterior wall of the ACE (FIGURE 1) (1). The iris diaphragm is divided into two major areas, namely the central pupillary zone and the outer ciliary zone (75). The edge of the former constitutes the boundary of the pupil. The root of the latter connects to the sclera and ciliary body (1, 75). The two zones are separated by the thickest region collarette, which is usually insensitive to pupil dilation and less affected by the eyelashes and eyelids (FIGURE 1) (1, 75). Therefore, the deliberate placement of transplants in the collarette region and nearby can improve specimen stabilization. It can also increase specimen accessibility by a microscope since the objective cannot satisfactorily reach transplants engrafted in the outer ciliary zone. The iris can be divided into the anterior border, stromal, muscular, anterior pigment epithelium, and posterior pigment epithelium layers in order from anterior to posterior (1, 75).

The stromal and muscular layers are made from fibrovascular tissue, sphincter, and dilator muscles and nerves (FIGURE 1) (1, 75). This makes the iris a highly vascular structure where the anterior ciliary and long posterior ciliary arteries merge into the major arterial circle at the root of the iris (FIGURE 1*D*). This major circle gives off numerous converging branches in the iris to the pupillary margin forming the minor arterial circle (76–79). The iris blood vessels are organized into anterior capillary, arteriovenular, and posterior capillary layers (78, 80). The anterior capillary layer contains plenty of thin, tortuous capillaries at a high density. The arteriovenular layer is localized in the iris stroma consisting of arteries and veins. The arteries show a bent spiral course and form an incomplete circle, i.e., the minor arterial circle of the iris, near the pupillary margin (FIGURE 1*D*). By contrast, the veins are straighter. They travel toward the root of the iris and drain into the tributaries of the vortex veins. The posterior capillary layer runs near dilator muscles (78, 80). Importantly, iridic blood vessels can effectively grow at a satisfactory rate into grafts at the iris. Therefore, the iris serves as an optimal transplantation site for small grafts like islets without being surgically reconnected to the recipient blood supply.

The iris adjusts the size of the pupil opening and thus controls the amount of light reaching the retina (1). It can do so because of two sets of iridic smooth muscles, i.e., the sphincter and dilator muscles. The former is shaped as an annular band and encircles the pupil contracting it in a circular motion. The latter is composed of radially oriented muscle fibers that fuse with the sphincter muscle in the pupillary zone and peripherally attach to the ciliary body. Contraction of the dilator muscle pulls the iris radially thereby enlarging the pupil (1). Contraction and dilation of the iridic smooth muscles are under strict control of parasympathetic and sympathetic innervation (FIGURE 4) (81). Parasympathetic postganglionic fibers, arising from the ciliary ganglion, in the short ciliary nerves innervate the sphincter muscle through muscarinic acetylcholine receptor signaling (FIGURE 4) (81). Therefore, muscarinic receptor antagonists such as atropine or tropicamide induce mydriasis, while muscarinic receptor agonists, such as pilocarpine, and the cholinesterase inhibitor physostigmine elicit miosis (82, 83). In the long ciliary nerves, sympathetic postganglionic fibers from the superior cervical ganglion synapse to the dilator muscles via noradrenaline α-adrenoreceptor communication (FIGURE 4) (81). α-Adrenoreceptor antagonists such as dapiprazole or thymoxamine trigger miosis, whereas the nonspecific adrenoreceptor agonist phenylephrine produces mydriasis (82, 83). In fact, both iridic parasympathetic and sympathetic nerve fibers integrate with grafts such as islets and can extend into and innervate islets placed onto the iris in a similar way to iridic blood vessels (30). Of note, such innervation can also provide intracameral islets with classical neurotransmitters, neuropeptides, and growth factors profitable for their immunotolerance (see sect. 3.4. for details), survival, and function (6, 30, 84). This adds a bonus to the ACE as a transplantation site.

### 2.3. Aqueous Humor

The AH is a transparent watery fluid that is continually secreted by the nonpigmented epithelium lining ridge-like extensions of the ciliary body known as the ciliary processes (FIGURE 1*F*) (1, 85). It pours into the narrow space between the posterior iris and the anterior lens, namely the posterior chamber of the eye, and then flows through the pupil into the ACE (FIGURE 1*F*). From there, AH leaves the eye in a passive manner via the conventional and nonconventional routes located in the iridocorneal angle (FIGURE 1*F*). The former is responsible for ∼90% of the drainage of AH, which passes sequentially through the trabecular meshwork, the inner wall of Schlemm’s canal and lumen, draining collector channels, aqueous veins, and episcleral veins, being driven by the intraocular pressure. The latter accounts for ∼10% of the drainage of AH that flows into the uveal meshwork, across the anterior face of the ciliary muscle, into the connective tissue between the muscle bundles, through the suprachoroidal space, and out through the sclera. This route is relatively independent of intraocular pressure (1, 85). The AH fills the ACE and the posterior chamber of the eye (FIGURE 1*F*). In these two chambers, the production of AH is delicately coupled with the drainage of AH through the conventional route to maintain intraocular pressure (15 mmHg) at a level above atmospheric pressure that helps the eyeball to keep its shape (86, 87). Caution should be exercised with regard to the volume and speed of intracameral injection so that intraocular pressure is kept around 15 mmHg. Importantly, the AH exhibits extraordinary immunomodulatory activities (see sect. 3.3. for details) (FIGURES 3 AND 4) (1, 85).

A contact lens to noninvasively and longitudinally measure the intraocular pressure in the rat ACE transplanted with islets has been developed (88). It revealed that intraocular pressure slightly increased initially but returned to normal soon in the early posttransplantation period. The animals whose ACE was engrafted with islets did not suffer from glaucoma (88). The slightly and transiently increased intraocular pressure did not influence the survival of intracameral islets. Until now, there has been no published study on the intraocular pressure in the human ACE engrafted with any tissues.

The AH is a slightly alkaline liquid resembling blood plasma in composition but contains less proteins and glucose and more lactic acid and ascorbic acid (1, 85). It has been reported that the mean plasma and aqueous glucose levels were 5.8 and 3.2 mM in nondiabetic subjects and 14.2 and 7.8 mM in patients with diabetes, respectively (89). Relatively low glucose levels in the AH bring about less metabolic stress on transplanted islets leading to survival benefits, especially before full vascularization. AH not only provides nutrients to eye tissues that lack a direct blood supply, such as the cornea and lens but also removes waste products from these tissues (1, 85).

The AH appears to contain relatively high levels of oxygen (90–93). As a matter of fact, the level of oxygen in the human ACE is competent enough to germinate a plant seed that was accidentally introduced there (94). In general, oxygen tension in the AH is higher than that in the interstitial fluid in different tissues and organs and comparable to that in the arterial blood (90–93). However, results obtained from different species with different techniques are somewhat contradictory (93). Three-fourths and one-fourth of aqueous oxygen tension come from the arterial blood through capillary walls and from the atmosphere across the cornea, respectively (93). Interestingly, aqueous oxygen tension in human subjects during local anesthesia and inhalation of 21% oxygen is significantly higher than simultaneously measured arterial blood oxygen tension (112.3 ± 6.2 mmHg vs. 85.7 ± 7.9 mmHg) (93). In such an oxygen-rich milieu, transplants survive and function better than in other transplantation sites (4, 23, 28–30, 84, 95). Interestingly, aqueous oxygen is not evenly distributed in the ACE (96). The highest oxygen level appears near the iris vasculature and the inner surface of the central cornea, whereas the lowest one occurs near the ACE angle and close to the lens in healthy rabbits (96). This suggests that transplants placed onto the central pupillary zone of the iris can gain more oxygen having higher viability and better function than those at the outer ciliary zone. However, a study in patients with cataracts showed that the highest and lowest levels of aqueous oxygen tension were detected at the ACE angle and at the center of the pupil (97). Unfortunately, the distribution of oxygen tension in different areas of the ACE of mice and in particular immunodeficient ones that are the most commonly used animal models for transplantation research has not been characterized.

**FIGURE 5.**
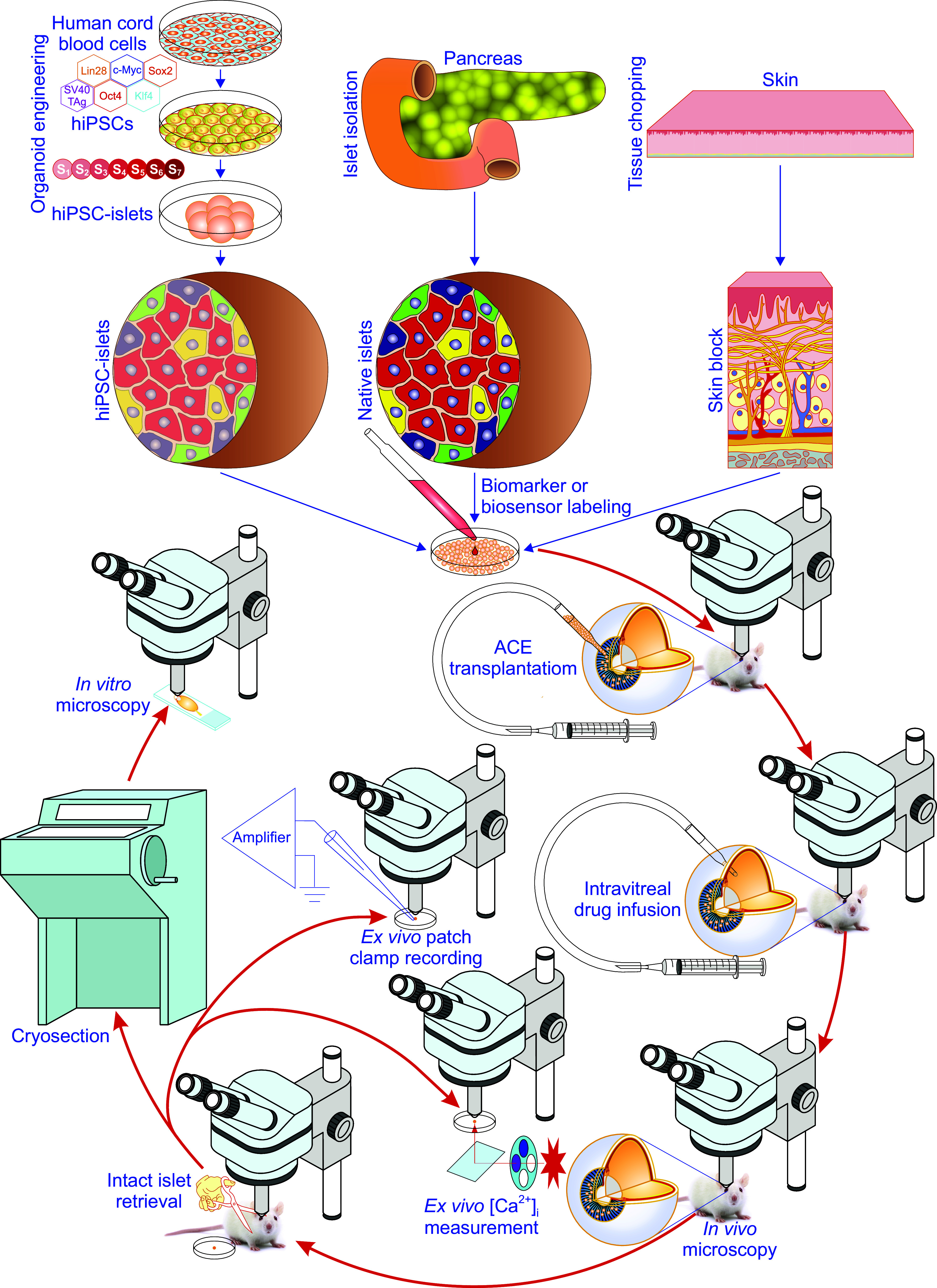
Methodological details of the anterior chamber of the eye (ACE) technology. Experimentally, the ACE technology can be carried through in the following order (4–7, 36, 63). First, different grafts like in vitro-engineered islets from human induced pluripotent stem cells (hiPSCs), isolated native islets from pancreata, and finely chopped skin are prepared and tested for quality in vitro. Second, grafts are genetically labeled with biomarkers or biosensors. This step is only necessary for intravital microimaging. Third, grafts are transplanted into the ACE. After the above 3 steps, there are multiple options including intravital microscopy, intravitreal drug infusion, intact retrieval of intracameral grafts for ex vivo cytoplasmic-free Ca^2+^ concentration ([Ca^2+^]_i_) measurement, ex vivo patch-clamp recording, and histological labeling/in vitro microscopy (4–7, 36, 63).

From the perspective of islet transplantation for in vivo imaging, intervening, and harvesting of single intact islet grafts, the AH is important. This is because it cannot only keep intracameral islets separate to prevent them from being aggregated, but can also accept and dissolve different reagents in the locally limited space of the ACE (63). The nonaggregated single intracameral islet grafts are best suited for in vivo imaging, easily reached by different reagents in a well-controlled manner with little or no systemic side effects and possibility for intact surgical retrieval with negligible mechanical stress and no chemical insult (FIGURE 5) (63). The retrieved grafts can directly be used for different functional assays such as measurements of cytoplasmic-free Ca^2+^ concentration ([Ca^2+^]_i_) and patch-clamp recordings without further cultivation therefore preserving in vivo gained phenotypes (FIGURE 5) (63).

## 3. OCULAR IMMUNE PRIVILEGE

The concept of immune privilege was created by Medawar and colleagues (38, 40) in the 1940s although the phenomenon of immune privilege was first observed in 1873 (3). This observation showed that homologous lip mucosa transplanted into the ACE of dogs and rabbits underwent prolonged survival and progressive growth, turning into a tumor-like mass (3). Unfortunately, the unavailability of theoretical and practical knowledge of immunology at that time did not allow a mechanistic explanation of the phenomenon. Even afterward, there was no attempt to elucidate the relationship of this phenomenon with immunity for more than 70 years, during which time immunological knowledge was gained (38–41, 45, 46, 48–50, 52, 54, 56, 98, 99). It was not until the 1940s that ground-breaking investigations were carried out regarding the reasons why grafts of foreign cells/tissues/organs undergo acute rejection (38–40). They demonstrated that skin transplants among genetically different individuals of the same species were exclusively rejected and convincingly attributed such rejection to the immune system of the recipient, which recognizes foreign molecules on the allogeneic skin and destroys them. Correspondingly, they termed these foreign molecules “transplantation antigens.” This elegant observation was recapitulated in many other situations where different cells/tissues/organs were grafted into a wide range of conventional body sites (38–40). In almost all locations throughout the body, the immune system recognizes different transplants carrying foreign antigens and attacks them relentlessly. Therefore, immune rejection of grafts in conventional body sites among genetically different individuals has been regarded as a classic transplantation dogma. Prolonged survival of allogeneic skins occurred due to the unique anatomical and immunological properties of the eye and brain. On the basis of these seminal contributions, the important transplantation term “immune privileged site” was coined, and the concept of immune privilege was created (FIGURE 3) (38, 40).

**FIGURE 6.**
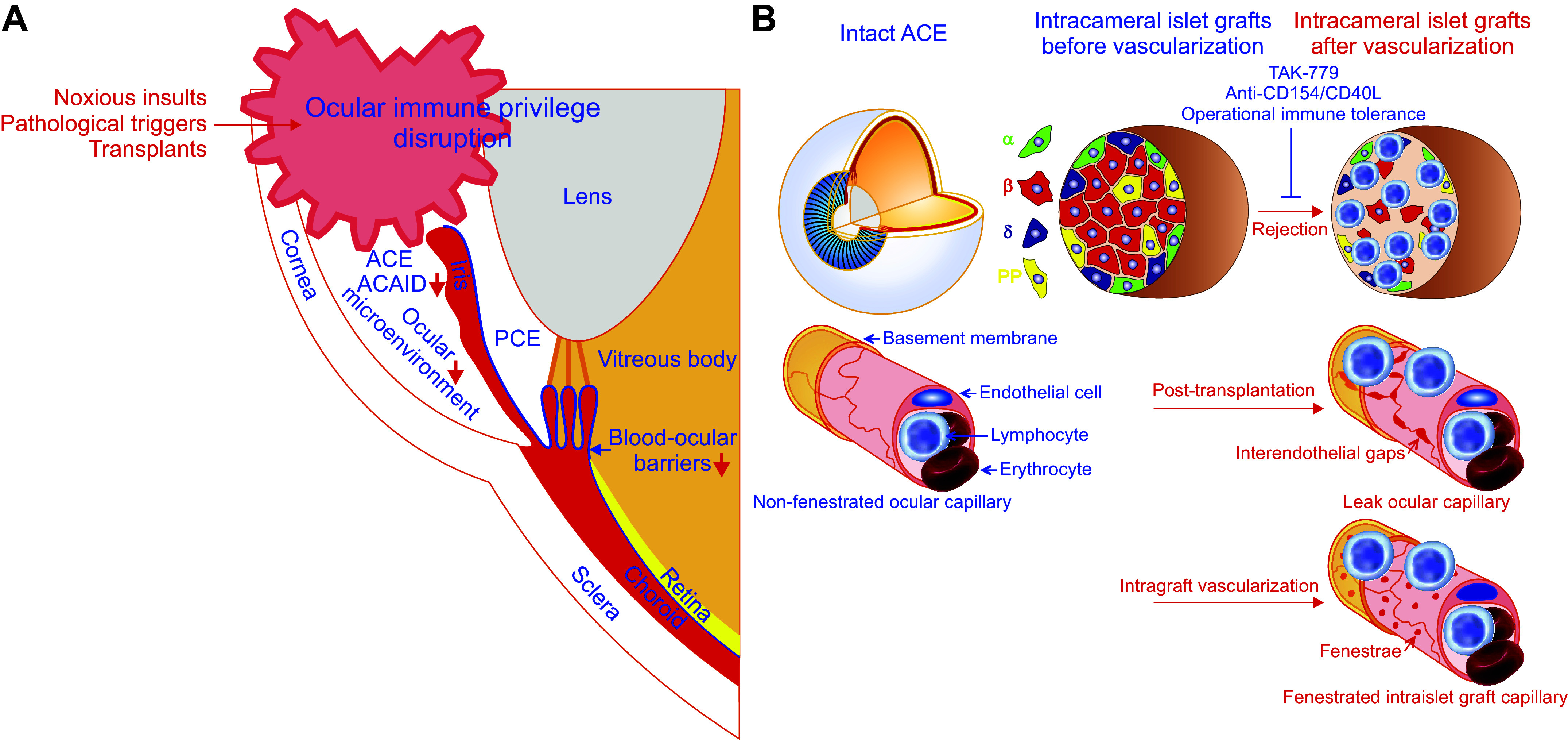
Ocular immune privilege disruption, intracameral alloislet rejection, and tolerance induction. *A:* the ocular immune privilege can be disrupted by noxious insults, pathological triggers, and transplants, which cause the breakdown of the blood-ocular barrier due to inflammation, ocular neovascularization or transplant revascularization, impair anterior chamber-associated immune deviation (ACAID) induction, and derange ocular microenvironment. *B:* intracameral alloislet rejection occurs alongside ocular immune privilege disruption induced by islet transplants, which can change nonfenestrated ocular capillaries into leak ones with interendothelial gaps and undergo vascularization characterized by fenestrated intraislet graft capillaries during posttransplantation (29). This alllorejection can be satisfactorily intervened by blocking T-cell chemokine receptors CCR5 and CXCR3 with TAK-779 or the binding of CD154/CD40L to CD40 primarily expressed on activated T cells with anti-CD154/CD40L blocking antibody, the latter reflecting induction of operational immune tolerance (22, 114). ACE, anterior chamber of the eye; PCE, posterior chamber of the eye; PP, pancreatic polypeptide.

The existence of ocular immune privileged sites is attributed to a diverse range of ocular and systemic mechanisms, which can be divided into three main layers (FIGURE 3) (38, 40–57, 98–112). The first one is the sequestration of transplantation antigens from the recipient’s immune system. Efficient blood-aqueous barriers and lack of efferent lymphatics isolate intraocular compartments from the systemic immune system by preventing free entry and exit of cells and some macromolecules into and out of the ACE (FIGURE 3) (38, 40). The second one is ACE-associated immune deviation (ACAID) (FIGURE 3). In fact, the ocular immune privilege does not solely rely on passive sequestration but is also actively regulated in response to antigenic challenges (41, 44–46, 48, 57, 98, 99, 101–107, 113). The third one is ocular microenvironment (FIGURE 3). Ocular cells like pigmented epithelia and parenchymal cells of the iris and ciliary body secrete immunosuppressive factors, such as transforming growth factor-β (TGF-β) and CD86, and also use contact-dependent mechanisms creating the immunosuppressive microenvironment in the ACE (FIGURES 3 AND 4) (43, 49–52, 56). In addition, neural inputs and light exposure also participate in the regulation of ocular immune privilege (FIGURE 4) (47, 54, 112). Ocular immune privilege disruption and restoration can happen under different scenarios (FIGURE 6) (22, 38, 114–122). The anatomical, cellular, and molecular features and their complex combinations underlying ocular immune privilege have over the years attracted a lot of attention (41–57, 98–112). These aspects of ocular immune privilege are detailed below.

### 3.1. Anatomical Basis

The ACE and some of its building components share unique anatomical features including the absence of lymphatic drainage and the existence of blood-ocular barriers and have formally been recognized as immune-privileged sites and tissues (FIGURES 3 AND 4) (57, 123–125). This led to the antigen sequestration paradigm for ocular immune privilege (38, 40, 48–50, 57, 109, 123, 124, 126, 127).

#### 3.1.1. Characteristics of local blood circulation and lymphatic drainage.

Medawar and colleagues (38, 40) believed that the eye lacks direct lymphatic drainage, and thus antigens and antigen-presenting cells could not move from the interior of the eye to the regional lymph nodes resulting in sequestration of antigen and antigen-expressing tissues. This original mechanism underlying ocular immune privilege emerged as a key milestone in the history of transplantation immunology, although it is now known that such a passive mechanism alone does not account fully for ocular immune privilege (38, 40, 41, 45, 46, 48–50, 52, 54, 56, 98, 99). Later on, lymphatic drainage was actually revealed in the ACE but significantly less than that in other body regions (46, 128–131). In addition to the deficiency of lymphatic drainage, nonfenestrated blood vessels also characterize the anatomy of the iris and the retina (46, 124, 132, 133). Quite a few blood vessels in the anterior segment of the eye are nonfenestrated and limit the extravasation of macromolecules and leukocytes from the blood into the ACE (FIGURE 6*B*) (46, 124, 132). Furthermore, some parts of the eye, including the cornea and lens that focus and transmit light, have no vessels (134–137). Apparently, these avascular structures disable access to immunogenic macromolecules and immune cells in the blood.

#### 3.1.2. Blood-ocular barriers.

The eye is equipped with complex anatomical entities to prevent free diffusion of blood solutes from the intravascular space into ocular tissues and compartments, thereby maintaining ocular immunosuppressive microenvironment (FIGURE 3) (48–50, 57, 109, 123, 124, 126, 127). Such entities are collectively termed blood-ocular barriers, mainly including the blood-retinal barrier and the blood-aqueous barrier (123, 124, 126, 127). The concept of the blood-aqueous barrier was first inferred from biochemical observations that plasma-derived proteins in the AH were reduced compared to proteins measured in the plasma and later proved anatomically (123, 124, 127). No anatomical barrier has been verified either between the posterior chamber and the vitreous humor or between the vitreous humor and the retina (124, 138). This is because the vitreous membrane is porous and allows a relatively free exchange of water-soluble compounds between the anterior, vitreous, and posterior chambers (FIGURE 1*F*) (124, 138). Indeed, the blood-retinal barrier protects the retina from being invaded by bloodborne pathogens. However, this beneficial role can be problematic. For example, it prevents systemically administrated drugs from reaching the diseased retina. This problem can be partially solved by making use of the ACE. Drugs or active biomolecules released from synthetic microcontainers or cell/tissue grafts placed into the ACE can pass through the porous vitreous membrane to reach the retina affected by diseases like vasoproliferative retinopathies (FIGURE 1*F*) (139–141).

The development of advanced electron microscopy and the discovery of suitable vascular tracers have successfully revealed ultrastructural details of the blood-ocular barriers (123, 124, 126, 127). In general, these barriers are attributed to tight junctions between ocular epithelial cells and between vascular endothelial cells (123, 124, 126, 127, 142–144).

The blood-aqueous barrier is situated in the anterior segment of the eye and can be separated into anterior and posterior parts, which restrict penetration of blood solutes into the posterior chamber and the anterior chamber, respectively (123, 124, 127, 138). Ultrastructural studies reveal that the blood-aqueous barrier is formed by tight junctions between the nonpigmented ciliary epithelial cells as well as those coupling vascular endothelial cells in ciliary processes, in the iris vasculature, and in the inner wall endothelium of the Schlemm’s canal (123, 124, 127, 138). In addition, pigmented epithelial cells on the posterior surface of the iris also contribute to the establishment of the blood-aqueous barrier. They are interconnected with one another through tight junctions to prevent proteins in the iris stroma from diffusing into the posterior chamber (127, 145). It has been proposed that tight junctions interconnect pigmented epithelial cells beneath both the photoreceptor cells of the retina and the secretory epithelial cells of the ciliary body as well as on the posterior surface of the iris, thereby forming a virtually continuous cell layer as an immunologic barrier for the eye (FIGURE 3) (48, 57).

Great attention has been paid to the molecular anatomy of blood-ocular barriers (123, 126, 142–144). The obtained results demonstrate that tight junctions as the actual component of blood-ocular barriers consist of tight junction proteins including the transmembrane protein junctional adhesion molecules, claudins and occludins, and the cytosolic proteins zonula occludens and cingulin (123, 126, 142–144). The former interact with one another in adjacent cells to hold them together, whereas the latter serve as bridges between transmembrane tight junction proteins and actin cytoskeleton by binding to them in series (123, 142, 143). Thus tight junctions enable blood-ocular barriers to restrict the penetration of blood solutes through the paracellular pathway (123, 126, 142–144).

Interestingly, epithelial cells not only build up various barriers, like the blood-ocular barriers, to separate different biological compartments in the body, but also have specific routes, e.g., receptor-mediated transport or transcytosis, to transport macromolecules between these compartments (146, 147). In-depth understanding of what extraocular regulatory factors and hormones can cross ciliary body epithelial cells, which form part of the blood-aqueous barrier, via receptor-mediated transport has the potential to unveil the pathogenesis of a range of eye diseases such as glaucoma. This may even lead to development of novel treatments for these eye diseases.

Of note, ample evidence has suggested that blood-ocular barriers are attained and sustained actively rather than passively and that ocular immune privileged sites are not fully sequestrated from the immune system (41, 42, 44–46, 48, 50, 57, 98, 99, 102–104, 112, 148–158).

### 3.2. Anterior Chamber-Associated Immune Deviation

The aforementioned anatomical uniqueness of the eye enabled the creation of a passive antigen sequestration paradigm for illustrating ocular immune privilege (38, 40, 48–50, 57, 109, 123, 124, 126, 127). Such a paradigm has remained unchallenged for more than two decades (38, 40, 148, 149). Eventually in the late 1970s, a new era in immune privilege research was put forward. Kaplan and colleagues (148, 149) carefully reexamined this paradigm by injecting allogeneic lymphoid cells into the rat ACE. They found that alloantigens associated with the injected cells escaped from the ACE and were detected by the systemic immune apparatus rather than completely sequestrated at the injection site (148, 149). Importantly, these alloantigens induced antigen-specific downregulation of cell-mediated immune responses and a simultaneous upregulation of the humoral immune response. Such deviant alloimmune phenotypes were referred to as “lymphocyte-induced immune deviation” (148). Subsequently, Niederkorn and colleagues (150) showed that these phenotypes were lymphoid cell type-independent, but critically depended on the placement of the alloantigens, namely in the anterior chamber of the eye. Therefore, they coined the new term “anterior chamber-associated immune deviation (ACAID)” instead of the original one “lymphocyte-induced immune deviation” to emphasize the critical role of the ACE in the induction of ACAID (FIGURE 3) (148, 150). As a deviant, antigen-specific systemic immune response to antigens placed into the ACE, ACAID is phenotypically characterized by an antigen-specific suppression of T_H_1 immune responses, such as delayed-type hypersensitivity (DTH) and complement-fixing antibodies, and concomitant stimulation of noncomplement-fixing IgG1 antibodies and cytotoxic T lymphocyte responses (41, 44–46, 48, 57, 98, 99, 102, 112, 148–150). ACAID also suppresses T_H_2-mediated immune responses, like allergic inflammatory lung diseases (41, 151). In addition, it has been demonstrated that ACAID is a stereotypic immune response that occurs when virtually any antigen is inoculated into the ACE (FIGURE 3) (45, 150). In fact, ACAID has been verified with ocular inoculation of a wide variety of antigens, including viruses, haptenated cells, soluble proteins tumor antigens, and histocompatibility antigens (FIGURE 3) (45, 98, 150, 152–157, 159, 160). Hence, the remarkable observations have resulted in a new immunological concept, namely active ocular immune privilege accounted for by ACAID (FIGURE 3) (41, 44–48, 57, 98, 99, 102–104, 106, 112, 148–150). ACAID has been mechanistically dissected (FIGURE 3). The obtained results demonstrate that ACAID is a complex dynamic immunoregulatory process involving multiple organ systems, including the ACE, thymus, spleen, and the sympathetic nervous system as well as various cell populations, e.g., F4/80+ antigen-presenting cells (APCs), natural killer (NK)1.1+ T cells, CD4+ regulatory cells, and CD8+ T cells (FIGURE 3). These components actively participate in the induction and maintenance of ACAID (FIGURE 3) (41, 42, 44–48, 50, 53, 57, 98, 99, 102–104, 106, 112, 148–150, 158).

#### 3.2.1. Ocular phase of ACAID.

ACAID is initiated as soon as alloantigens enter the ACE and then proceeds through its ocular phase (FIGURE 3) (41, 42, 46, 48, 57, 98, 99, 102, 150). Importantly, the ACE is not only the starting point of ACAID but also plays an obligatory role in ACAID development (98). Hence, premature removal of the eye subjected to alloantigen inoculation aborts the development of ACAID (161–163). A mechanistic explanation for this obligatory role is that the ACE serves as a depo from which small quantities of antigens escape slowly and continuously into the blood (164, 165). Within the ACE, alloantigens are captured, processed, and presented sequentially by ocular F4/80+ APCs under the coordination of a series of AH cytokines and chemokines among which TGF-β is critical (FIGURE 3) (41, 98, 166–168). Following exposure to TGF-β, ocular APCs become different from conventional APCs and display a reduced synthesis of T_H_1-inducing cytokine IL-12, an increased production of cytokine IL-10, a decreased expression of CD40 costimulatory molecule, and the autocrine production of TGF-β (FIGURE 3) (41, 98, 169–171). Meanwhile, these indigenous APCs in the ACE also gain the capacity to produce the potent chemokine macrophage inflammatory protein-2, which acts as a key player in the splenic phase of ACAID (see below for details) (FIGURE 3) (41, 172). Furthermore, apoptosis of antigenic cells and in particular apoptosis induced by Fas ligand downstream of tumor necrosis factor-α are crucial for antigen processing during induction of ACAID (41, 163, 173–175). In addition, ligation of the complement 3b receptor on the surface of F4/80+ ocular APCs critically contributes to the induction of ACAID (41, 176). Following ocular antigen processing, F4/80+ ocular APCs enter the blood stream and migrate to the thymus and spleen (FIGURE 3) (41, 42, 44, 98, 99). The APCs have been isolated from blood at 48 h postinoculation and are able to induce ACAID when transferred to other recipients (41, 160, 177). The APCs must express F4/80 and the major histocompatibility complex class I-like molecule CD1d to execute the subsequent cellular interactions in the thymic and splenic phases (41, 178, 179).

#### 3.2.2. Thymic phase of ACAID.

Following exposure to antigens in the ACE, the primed F4/80+ ocular APCs exit through the ocular trabecular meshwork into the bloodstream and then flow into the thymus to continue inducing ACAID (FIGURES 1*F* AND 3). The thymus has been demonstrated to play an essential role in the induction of ACAID (FIGURE 3) (41, 180). Wang et al. (160, 180) have shown that ACAID does not arise in mice subjected to thymectomy followed by intracameral inoculation of alloantigens or subsequent to intravenous transfusion of antigen-pulsed F4/80+ ocular macrophages that invoke ACAID in euthymic animals. During the thymic phase of ACAID, the key event that takes place is that F4/80+ ocular APCs bring about the generation of CD4-CD8-NK1.1+ thymocytes that move into circulation and home to the spleen, where they contribute to the production of splenic suppressor T cells (FIGURE 3) (41, 98, 160, 181).

#### 3.2.3. Splenic phase of ACAID.

Following exposure to alloantigens inoculated into the ACE, the primed F4/80+ ocular APCs escaping into the bloodstream are destined not only for the thymus as aforementioned but also preferentially for the spleen (FIGURE 3) (41, 57, 98, 99, 180, 182). Importantly, the latter branch appears to be the primary route of migration of F4/80+ ocular APCs and thus being highlighted as the camero-splenic axis of ACAID (FIGURE 3) (41, 57, 98, 99, 180, 182). The intact spleen is required for the induction and expression of ACAID (41, 57, 98, 99, 182). This is because splenectomy abolishes the induction and expression of ACAID during the first 7 days following ocular inoculation of alloantigens (182). The splenic phase of ACAID is initiated by the migration of F4/80+ ocular APCs to the spleen (FIGURE 3) (41, 57, 98, 99, 182). This important phase appears as complex cell interactions governed by various cytokines and chemokines and culminates in the production of CD4+ afferent and CD8+ efferent suppressor cells, the former suppressing the induction of DTH responses whereas the latter inhibiting the expression of DTH responses, thereby ultimately resulting in ACAID (FIGURE 3) (41, 57, 98, 99, 182, 183).

Within the spleen, the primed F4/80+ ocular APCs, which express CD-1d and display activation of signal transducer and activator of transcription-6, recruit CD4+ NKT cells by releasing a potent chemoattractant macrophage inflammatory protein-2 (MIP-2; FIGURE 3). The recruited CD4+ NKT cells in turn secrete a chemokine called regulated on activation normal T-expressed and presumably secreted (RANTES) that attract additional cells into the marginal zone of the spleen where B cells, CD4+ NKT cells, CD4+ T cells, γδ T cells, and CD8+ T cells interact with each other in the dynamic niche composed of cytokines and chemokines (FIGURE 3) (41, 183). The complex cell interactions drive the differentiation of CD8+ T cells into end-stage ACAID Treg cells, so-called CD8+ efferent suppressor cells (FIGURE 3) (41, 183).

F4/80+ ocular APCs are inevitably subjected to ligation of the complement 3b (C3b) receptor on their surface as an obligatory event during the splenic phase of ACAID (41, 176, 183). This elevates IL-10 and TGF-β production and reduces IL-12 release from these APCs. With the guidance of increased IL-10 and TGF- β, the APCs process ocular antigens into antigenic peptides and release them into the marginal zone of the spleen (FIGURE 3). In addition to F4/80+ ocular APCs, B cells prerecruited in this zone appear to be obligatory for alloantigen processing in the spleen (FIGURE 3). They likely serve as another population of APCS to capture and internalize antigenic peptides released from F4/80+ ocular APCs via their antigen-specific B cell receptors and proliferate before presenting the reprocessed peptides to T cells (41, 183). The generation of the CD8+ end-stage ACAID Treg cells needs CD4+ T cells. Both of them must be exposed to ocular antigens presented by major histocompatibility complex (MHC) class II and MHC class I molecules, respectively, to become antigen-specific (183). In vitro evidence has suggested that B cells present both MHC class I-restricted and MHC class II-restricted antigens to CD8+ T cells and CD4+ T cells, respectively (183, 184). Furthermore, splenic γδ T cells are regarded as indispensable participants in the induction of ACAID (FIGURE 3) (41, 183, 185–188). They must have the capacity to produce IL-10 but do not act as ancillary APCs or as end-stage Treg cells although their precise role in ACAID is not known (FIGURE 3) (183, 188).

CD4-CD8-NK1.1+ cells generated in the thymus also move into the spleen to join complex cell interactions. In this context, they evoke the production of CD8+ efferent regulatory cells (FIGURE 3) (41, 98, 160, 181).

Eventually, CD4+ afferent and CD8+ efferent suppressor cells as well as other cell populations such as regulatory B cells and γδ Tregs resulting from the complex cell interactions in the marginal zone of the spleen spread systemically and induce ACAID (FIGURE 3) (41, 42, 57, 98, 99, 182, 183).

#### 3.2.4. Neural regulation of ACAID.

Sympathetic nerves densely innervate the eye, spleen, and thymus and are necessary for the induction of ACAID (FIGURES 1 AND 4) (6, 41, 183, 189). It has been found that the sympathetic nervous system regulates the induction of ACAID (41, 183). Depletion of ocular or systemic sympathetic innervation also brings about ACAID disruption that results in rapid rejection of tumor cells carrying minor histocompatibility antigens placed into the ACE or abolish the induction of splenic suppressor T cells following intracameral injection of trinitrophenol-bovine serum albumin (54, 190, 191). Chemical sympathetectomy prevents the induction of ACAID most likely due to the impaired generation of CD4+ NKT cells, which are required for the production of CD8+ end-stage ACAID Treg cells (191). However, such sympathetic ablation had no effect on the generation of ocular APCs (191). Collectively, sympathetic innervation appears to be crucial for the induction of ACAID but differentially influences different phases of ACAID (FIGURES 1 AND 4) (41, 183, 191).

The involvement of corneal sensory innervation in ACAID induction is verified by experimental evidence showing that ACAID cannot occur in eyes transplanted with allocorneas within the first 8 weeks posttransplantation, but indeed appears after 12 weeks posttransplantation (192). Furthermore, the transection of nerve axons on the corneal surface and stroma coming from the corneal limbus by making a circular, nonpenetrating incision along the corneal limbus prevents the appearance of ACAID induced by injection of bovine serum albumin into the ACE (192). These findings support that ocular sensory nerves are important for ocular immune homeostasis (FIGURES 1 AND 4).

#### 3.2.5. Regulation of ACAID by light.

Interestingly, exposure to visible light is essential for the induction and expression of ACAID (FIGURE 4) (47, 193, 194). Ferguson and coworkers (194) visualized that ocular inoculation of alloantigens cannot induce ACAID in mice raised in constant darkness or removed from light housing to darkness. They also verified that the induction of ACAID in mice exposed to the normal light-dark cycle depended on the presence of a specific wavelength of visible light (see sect. 3.5) (193, 194).

#### 3.2.6. Therapeutic potential of ACAID.

As end-stage ACAID Treg cells, CD8+ efferent suppressor cells not only suppress T_H_1 immune responses but also mitigate T_H_2-mediated inflammation (41, 42, 48, 57, 98, 99, 183). This raises therapeutic possibilities to prevent or treat some autoimmune and immunopathogenic diseases as well as allograft rejection by harnessing ACAID. So far, therapeutic interventions of some experimental inflammatory diseases, by inducing ACAID, have been applied to animal studies (151, 154, 195). Mizuno et al. (154) injected retinal soluble antigen (S-Ag) alone or together with complete Freund’s adjuvant into the rat ACE to evoke ACAID and subsequently applied a uveitogenic dose of S-Ag to the same animals for induction of experimental autoimmune uveitis. It turned out that in the majority of rats, their eyes were normal. Only a minority of rats suffered from a mild form of uveoretinitis. These findings verify that ACAID induced by intracameral inoculation of S-Ag can effectively mitigate the development of experimental autoimmune uveitis, thereby preserving the retina in rats (154). In a mouse model of rheumatoid arthritis induced by type II collagen (CII), intracameral injection of CII or adoptive transfer of either in vitro-generated CII-specific ACAID macrophages or CII-specific in vitro-generated T-regulatory cells resulted in the reliable elicitation of ACAID. This significantly inhibits CII-induced ear swelling, serving as a marker for rheumatoid arthritis (195). Katagiri and coworkers (151) have examined the role of ACAID in the mitigation of T_H_2 cell-dependent allergic lung disease resulting from intratracheal challenge with ovalbumin in mice. They injected ovalbumin into the ACE or ovalbumin-pulsed ACAID-inducing APCs exposed in vitro to TGF-β2 into the veins of susceptible mice. As expected, treatments induced ovalbumin-specific ACAID and, importantly, prevented the animals from developing the typical signs of ovalbumin-dependent experimental allergic lung disease. This work demonstrates that ACAID also suppresses T_H_2-mediated inflammation (151).

Driven by the fact that the ACAID-inhibited T_H_1 and T_H_2 responses act as key events in allograft rejection, special attention has been paid to therapeutic manipulation of ACAID in promoting allograft survival (41, 42, 48, 49, 57, 98, 99, 150, 182, 183, 196, 197). Experimental evidence shows that intracameral inoculation of allogeneic cells significantly prolonged allogeneic skin graft survival in mice. This effect was intimately associated with alloantigen-specific ACAID and was abolished by splenectomy (150, 182). Studies on murine orthotopic corneal transplantation corroborate that alloantigenically specific ACAID plays a critical role in the success of grafted corneas. This is because orthotopic corneal allografts were accepted indefinitely by adult mice showing suppression of alloantigen-specific DTH, a hallmark of ACAID (197). These findings provide experimental evidence that manipulation of ACAID holds promising therapeutic potential for promoting allograft survival (150, 182, 196, 197). Inoculation of human leukocyte antigens (HLAs) from a donor in the ACE of a recipient patient could be one way to mitigate rejection of allografts subsequent to transplantation.

Overall, there is no doubt that ACAID constitutes a major component of ocular immune privilege (FIGURE 3). Harnessing ACAID holds great potential for preventing and treating some autoimmune and immunopathogenic diseases as well as allograft rejection.

### 3.3. Ocular Microenvironment

Somewhat penetrable blood-ocular barriers in and lymphatic drainage from the eye challenge the antigen sequestration paradigm (46, 57, 99, 128–131). This has created interest in exploring additional mechanisms behind ocular immune privilege (41, 45, 46, 48–50, 52, 54, 56, 98, 99). It turned out that the ocular immunosuppressive microenvironment serves as one of the most important mechanisms whereby immune privilege operates within the eye (FIGURES 3 AND 4) (45, 46, 48–50, 52, 54, 56, 98, 99). Such a microenvironment is bestowed with the power to counteract both the innate and adaptive immune responses thereby actively contributing ocular immune privilege (44). Such a microenvironment mainly consists of various immunosuppressive and anti-inflammatory factors, which are solubilized in ocular fluids or bound to cell surface membranes directly exposed to ocular fluids (FIGURES 3 AND 4) (45, 46, 48–50, 52, 54, 56, 98, 99).

Among ocular fluids, AH has attracted the most attention and has been found to possess abilities to modulate various immune activities. It reduces the proliferation of mitogen and antigen-stimulated T cells in vitro and inhibits DTH responses mediated by alloimmune T_H_1 cells when being introduced into the ACE (198, 199). High-pressure liquid chromatography (HPLC) revealed that the anti-proliferative activity of AH is attributed to two HPLC fractions, one mainly consisting of 25-kDa molecules and the other containing components with molecular mass < 5 kDa (113, 200). Both fractions showed potent activities against the proliferation and IL-2 production by alloantigen-activated lymphocytes (200), whereas only the low molecular mass fraction inhibited the proliferation of thymocytes, i.e., immature T cells, following stimulation with IL-1 and tumor necrosis factor-α (TNF- α) (113). The high molecular mass fraction accommodated TGF-β2 and the low one contained a set of neuropeptides as actual immunosuppressive players (113, 200–203). Within AH, a range of soluble immunosuppressive and anti-inflammatory molecules that counteract both the innate and adaptive immune responses, which can cause irreparable ocular damage, have been characterized (44). In essence, these molecules are cytokines, chemokines, lymphokines, or neuropeptides (FIGURES 3 AND 4) (45, 46, 48–50, 52, 54, 56, 98, 99). They come into play by orchestrating a variety of singling pathways (45, 46, 48–50, 52, 54, 56, 98, 99).

In addition, proinflammatory cytokines and chemokines can also appear in the ocular environment under proinflammatory conditions including allotransplantation. In this situation, corneal endothelial cells, iris pigment epithelial cells, ciliary body pigment epithelial cells, and retinal pigment epithelial cells suppress the activation of bystander CD4+ T cells by proinflammatory cytokines and chemokines to maintain ocular immune homeostasis and limit inflammation (204).

#### 3.3.1. Immunosuppressive and anti-inflammatory molecules mitigating innate immune responses.

To mitigate the innate immune response in the ACE, a diversity of anti-inflammatory and immunosuppressive cytokines as well as free radical scavengers are produced in AH (FIGURE 4) (205). Interestingly, some factors in the AH can purge inflammatory cells by promoting apoptosis of neutrophils and macrophages that participate in inflammatory processes without influencing ocular tissues, such as the corneal endothelium and iris/ciliary body (206). Activation of the alternative pathway of complement by bacterial products as a part of the innate immune response not only results in the production of a membrane attack complex that perforates the plasma membrane leading to osmotic lysis of both bacterial and mammalian cells but also induces generation of soluble factors that recruit and activate neutrophils. Such an innate immune response is counteracted by complement regulatory proteins (CRPs) that are present in the AH and coat the membranes of cells lining the ACE (FIGURE 4) (207–211). As part of the innate immune system, NK cells can recognize and kill cancer cells and virus-infected cells immediately without having encountered them before. However, this can hardly happen in the eye due to two major reasons (212). One is that the AH contains macrophage migration inhibitory factor and TGF-β that produce immediate and delayed inhibition of NK cell activity, respectively (FIGURE 4) (212). The other is that nonclassical MHC class Ib molecules such as human leukocyte antigen E (HLA-E) in humans and Qa-2 in mice expressed by corneal endothelial cells are able to convey “off” signals to NK cells (FIGURE 4) (212, 213). In the eye, α-melanocyte stimulating hormone (α-MSH) and neuropeptide Y (NPY) can activate M1 macrophages to remove harmful materials thereby preventing inflammation (FIGURE 4) (56, 214, 215). The glycoprotein pigment epithelium-derived factor as a member of the family of noninhibitory serpins contributes to innate immune privilege of the eye by suppressing lipopolysaccharide-driven macrophage activation (FIGURE 4) (216). These findings demonstrate that AH-borne factors against the innate immune responses are important to the immune privilege of the ACE (FIGURE 4).

#### 3.3.2. Immunosuppressive and anti-inflammatory molecules alleviating adaptive immune responses.

The adaptive immune response is injurious to the eye but significantly compromised because of complex ocular immune privilege. As part of ocular immune privilege, various immunosuppressive and anti-inflammatory factors blocking the adaptive immune response are present in the AH and are more complex than those counteracting the innate immune response (FIGURE 4) (44).

In a repertoire of soluble factors that block the adaptive immune response in the eye, at least five of them including TGF-β, α-MSH, vasoactive intestinal peptide (VIP), calcitonin gene-related protein (CGRP), and somatostatin inhibit the expression of T-cell-mediated inflammation (FIGURE 4) (113, 200, 201, 203, 217). The inhibitory effect of somatostatin on the adaptive immune response in the ACE suggests that intracameral islet grafts containing somatostatin-secreting δ cells have a better survival rate than other grafts (FIGURE 4). Furthermore, indoleamine dioxygenase (IDO) produced by cells lining the ACE catabolizes tryptophan, a key amino acid necessary for T lymphocyte survival, and thus depletes tryptophan and induces T-cell apoptosis (FIGURE 4) (218, 219). Moreover, CRPs in the AH and expressed on the plasma membranes of many cells lining the interior of the eye play an important role in maintaining ocular immunological homeostasis (FIGURE 4). The fact that administration of neutralizing antibodies to CRPs caused spontaneous ocular inflammation makes one believe that CRPs can restrain the untoward effects of slight complement activation that occurs constitutively in the body, including the eye under normal homeostatic conditions (220). Soluble cytotoxic T-lymphocyte antigen-2α (CTLA-2α), thrombospondin-1 (TSP-1), and retinoic acid are able to activate TGF-β, resulting in upregulation of Treg cells that can suppress bystander effector T cells, thereby maintaining the immunosuppressive microenvironment in the eye (FIGURE 4) (53, 56, 221, 222). In the AH, corticosterone and hydrocortisone are present and execute their immunosuppressive action by suppressing TNF-α-induced thymocyte proliferation (FIGURE 4). In addition, cortisol-binding globulin is absent in the AH. This can enhance the immunosuppressive activity of the glucocorticoids (223).

The corneal endothelium, iris, ciliary body, and retina express a series of cell membrane-bound proteins that function as apoptosis inducers or proliferation inhibitors of T cells migrating into the eye (44). The type-II transmembrane protein Fas ligand (FasL) appears throughout the eye to purge activated T cells and neutrophils when they enter the eye where there are viral infections or foreign grafts (224–226). Programmed death-ligand 1 (PD-L1), a member of the B7 family of membrane proteins, is expressed in the corneal endothelial and stromal cells, iris, ciliary body, and retina to down-regulate T-cell proliferation and cytokine production and drive apoptosis of inflammatory cells (FIGURE 4) (227–233). This protein is needed for corneal allograft survival (227, 228). PD-L1 undergoes upregulation in the eyes where either sympathetic ophthalmia happens or proinflammatory cytokines such as TNF-α and IFN-γ appear. This indicates that PD-L1 can quench immune-mediated ocular inflammation (229). The TNF family member tumor necrosis factor-related apoptosis-inducing ligand (TRAIL) is localized and functions similarly to PD-L1 on the corneal endothelium, iris, ciliary body, and retina to contribute to ocular immune privilege (FIGURE 4) (234, 235).

Unlike most nucleated cells in the body, the corneal endothelium, the lens epithelium, and some retinal cells are devoid of MHC‐Ia molecules on their surface (46, 98, 236, 237). Therefore, these cells are resistant to MHC‐Ia-dependent/cytotoxic T lymphocytes (CTL)-mediated cytolysis. However, they are potentially vulnerable to NK cell-mediated damage. Cells lining the ACE and some retinal cells express nonclassical MHC class I (MHC‐Ib) molecules to inhibit NK cell cytotoxicity (44, 213, 238). Apparently, the altered expression of MHC antigens is a strategy for creating ocular immune privilege (44, 46, 98).

### 3.4. Neural Inputs

The eye is directly exposed to the environment, has limited regeneration capacity, and hardly recovers its full function from adverse incidents like noxious stimuli and uncontrolled inflammatory immune responses. As an evolutionary adaptation, the eye is richly innervated by sensory and autonomic nerves to quickly react to noxious stimuli and minimize their consequences (FIGURES 1 AND 4) (57, 81, 239). The ocular innervation not only provides reflex arcs for a quick escape from danger but also several neuropeptides that limit inflammatory immune responses, sustain the ocular immune privilege, and interact with other mechanisms, such as local TGF-β production and ACAID (FIGURES 1 AND 4; see sects. 3.2.4. and 3.3 for details), to maintain ocular immune homeostasis (FIGURE 4) (54, 112).

In the eye, autonomic nerves release NPY mainly through noradrenergic terminals and VIP, somatostatin, and CGRP dominantly through cholinergic endings (54, 81, 202, 240–242). Ocular sensory nerves also secret CGRP (243, 244). These four neuropeptides act as important players in the AH to maintain ocular immune privilege (FIGURE 4; sect. 3.3. for details) (44, 45, 49–52, 54, 56). Compared to the above-discussed neuropeptides, the classical neurotransmitters noradrenaline, adrenaline, and acetylcholine released from ocular autonomic nerves are much less understood with respect to their roles in the modulation of ocular immune privilege. A study shows that photopic light exposure elevated noradrenaline and adrenaline levels in the eye reduced the expression of α_1A_-adrenoceptor in the retina and protected the blood-retina barrier in mice with experimental autoimmune uveoretinitis (245). Of note, sympathetic and parasympathetic nerve terminals release the classical neurotransmitters noradrenaline/adrenaline and acetylcholine that act on adrenergic and cholinergic targets in immune cells to produce complex immunological effects outside the eye (246–249). Likewise, within the eye, there is not only dense ocular autonomic innervation that releases sufficient noradrenaline, adrenaline, and acetylcholine but also resident or infiltrating immune cells under physiological and pathological conditions due to the incomplete sequestration of the eye from the immune system (48, 57, 81, 104).

In fact, ocular sympathetic innervation also regulates the production of TGF-β, an important immunosuppressor, in the AH to support ocular immune privilege (190, 191). This was revealed by studies showing that surgical superior cervical ganglionectomy and systemic chemical sympathectomy with 6-OHDA effectively ablated ocular sympathetic innervation, significantly decreased active TGF-β levels in the AH, and abolished ocular immune privilege (190, 191). Moreover, corneal sensory nerves can also act to control TGF-β production in the AH for ocular immune homeostasis. This is evidenced by the fact that a rapid increase in active TGF-β levels occurred in the AH following scratching the central cornea with a syringe needle (112).

Both ocular sympathetic and corneal sensory innervations contribute to ocular immune privilege by regulating the induction and maintenance of ACAID (FIGURE 4; see 3.2.4 for details).

### 3.5. The Importance of Light in the Maintenance of Immune Privilege within the Eye

Light acts as a key player in maintaining ocular immune privilege (FIGURE 4). It has been found that intracameral Treg cells cannot be activated in dark-reared mice, light-reared ones placed in the dark, and light-reared ones whose eyelids were closed after their ACE received antigen inoculation (194). Visible light can directly activate intraocular Treg cells that cause systemic immune suppression. This light-induced intraocular condition can be eliminated by placing light-reared mice in the dark for 18 h after intracameral antigen inoculation and be induced in adult dark-reared mice by putting them back into the light for just over 24 h before intracameral antigen inoculation (194). The effective wavelengths of light that induce the intraocular immune reactions ranged between 500 and 510 nm (193). These findings demonstrate that light can directly induce ACAID and that light deprivation blocks the formation of Treg cells and abrogates the induction of ACAID following intracameral antigen inoculation.

Light-sensitive iridic sympathetic and parasympathetic nerve terminals contain and release the neuropeptide transmitters VIP and NPY into the ACE where they contribute to ocular immune privilege by mitigating immune responses (56, 81, 214, 215, 240–242). This appears to be an additional mechanism whereby light participates in the formation of ocular immune privilege.

It has been verified that exosomes produced by retinal pigment epithelial cells participate in blue-light photostimulation-induced ocular immune responses (250). Retinal pigment epithelial cells following blue-light photostimulation (488 nm for 6 h) released more exosomes containing IL-1β, IL-18, and caspase-1 and elevated NLRP3 inflammasome activity than control treatment (250). Furthermore, it has been suggested that retinal pigment epithelial cell-derived exosomes are involved at least in part in complement-driven innate immune responses in age-related macular degeneration (251). Taken together, it appears that light not only helps form ocular immune privilege but can also induce immune responses. Light with different wavelengths, intensities, and durations may cause distinct immunological effects.

### 3.6. Ocular Immune Privilege Disruption and Restoration

The eye is immunologically privileged but this privilege is not eternal and can increase, decrease, or even collapse under different conditions (FIGURE 6). For example, when the eye encounters noxious insults, pathological triggers, or transplants, ocular immune privilege disruption can occur (FIGURE 6). In essence, disruption of ocular immune privilege results from the breakdown of the blood-ocular barrier due to inflammatory insults, ocular neovascularization or transplant revascularization, impaired ACAID induction, and derangement of the ocular immunosuppressive environment (FIGURE 6). In fact, ocular immune privilege disruption accounts for a series of ocular diseases or intraocular transplant rejection. Owing to the importance of ocular immune privilege, considerable attention has been paid to its preservation and restoration. The obtained findings show that anti-inflammatory intervention, angiostatic therapy, and immunosuppressive reagent therapy are effective.

#### 3.6.1. Irritative and injurious insult-induced impairment of ocular immune privilege and its mitigation.

It has been shown that noxious stimulation of the corneal surface induces a local ocular stress response manifested by a breakdown of the blood-aqueous barrier, subsequent plasma protein leakage into the AH, and an increase in intraocular pressure (115). The immediate defensive response is nonimmunogenic and mediated by the ophthalmic division of the trigeminal nerve (115). This demonstrates that noxious stimulation is enough to break down the blood-aqueous barrier leading to aberrant ocular immune homeostasis (FIGURE 6).

Furthermore, a study reported that corneal neovascularization occurred as early as 3 days, peaks ∼2 weeks, and remains for quite a long time after the placement of three interrupted sutures in the central cornea (116). Concurrent with corneal neovascularization, corneal inflammation appears after 2 days, reaches its peak at 2 weeks, and becomes unobservable 4 weeks after suture placement. This inflammatory corneal neovascularization results in disruption of ocular immune privilege, demonstrated by the incapability of inducing ACAID from the first week of the neovascularization. Interestingly, early treatment with anti-inflammatory or angiostatic agents during the first 2 weeks after neovascularization induction regains the capability of inducing ACAID, reflecting recovery of ocular immune privilege from corneal suture, whereas delayed treatment does not. These findings demonstrate that inflammatory corneal neovascularization induced by irritative and injurious insult markedly disrupts ocular immune privilege (FIGURE 6*A*). Early anti-inflammatory interventions can effectively restore ocular immune privilege (116). Altogether these studies emphasize that the worse noxious insults the eye suffers from, the more severe disruption of ocular immune privilege occurs (FIGURE 6*A*). Therefore, one should minimize as much as possible invasive surgical procedures when placing transplants into the ACE.

#### 3.6.2. Disruption of ocular immune privilege in uveitis and its intervention.

A series of studies reveal that in eyes with uveitis, intraocular inflammation severely compromises ocular immune privilege in various ways (117–119). It breaks down the blood-ocular barrier by dilating the iris and ciliary body vessels. This allows inflammatory cells, mediators, and proteins to infiltrate into the usually immune-privileged intraocular environment to initiate and propagate autoimmune intraocular inflammation (118). In experimental autoimmune uveitis, a breach of the immunosuppressive ocular microenvironment in the AH happens even before the onset of the detectable intraocular inflammation (119). Such a deteriorated microenvironment can hardly support ACAID (117). Experimental autoimmune uveitis only lasts between 30 and 90 days and spontaneously resolves with a decrease in the proinflammatory cytokine IL-6 and an increase in the immunosuppressive factor TGF-β as well as restoration of ocular immune homeostasis (43, 119). According to the pathogenic mechanisms of uveitis, restoration of blood-ocular barriers with anti-inflammatory and angiostatic reagents has been one of the clinical therapies for treating patients with uveitis (118). Reestablishment of ocular immunosuppressive microenvironment by replenishing immunosuppressive factors like TGF-β and α-MSH shows great potential as a therapeutic strategy for uveitis treatment (120, 121).

#### 3.6.3. Allorejection and allotolerance induction in the ACE.

Like in other transplantation sites, allorejection happens in the ACE when intracameral grafts are vascularized. Noteworthy is, however, that intracameral allotolerance is inducible (FIGURE 6) (38, 252–255).

#### 3.6.4. Rejection of alloskin grafts in the ACE.

It has been found that small pieces of alloskin transplanted into the rabbit ACE are vascularized if they are attached to the iris, but not if they have no contact with the iris. Interestingly, intracameral allografts are rejected only if they are vascularized. Otherwise, they survive well. These findings point out that intracameral blood supply is not necessary for intracameral alloskin survival and importantly emphasize that the breakdown of blood-ocular barrier resulting from graft vascularization acts as a key player in the rejection of intracameral allografts (38).

#### 3.6.5. Orthotopic corneal allograft rejection and its tolerance induction.

It is well known that as an antigen-specific form of peripheral immune tolerance, ACAID not only contributes to the establishment and maintenance of ocular immune privilege to protect the eye but also acts as a mechanism of allotolerance induction to promote allotransplant survival in the eye and other orthotopic sites (41, 44–46, 48, 57, 98, 99, 102, 122, 150, 182). Murine models of allogeneic corneal transplantation show that most orthotopic corneal transplants can only survive for 20 days. Interestingly, intracameral injection of MHC-Ia-positive or -negative spleen cells or corneal endothelial cells into the left ACE significantly prolongs the survival of corneas transplanted orthotopically onto the right eyes and substantially reduces the rejection rate of these transplants (122). More interestingly, 60% and 90% of CB6F1 mice can permanently accept NZB mouse corneas transplanted onto their right eyes when their left ACEs are intracamerally inoculated with MHC-Ia-negative spleen cells and corneal endothelia, respectively, from NZB mice. Such tolerance induction occurs with induction of ACAID and dissipate when ACAID induction is blocked by splenectomy (122). These findings corroborate that intracameral inoculation with alloantigens critically relies on ACAID induction through the camero-splenic axis to effectively induce corneal transplant tolerance and suggests that this immunological manipulation can be used as a general approach to preventing intracameral allograft rejection (122).

#### 3.6.6. Dynamic rejection of and tolerance induction in intracameral alloislets.

Intracameral transplantation of alloislets is sufficient to derange ocular immune homeostasis resulting in their rejection in mice and baboons (FIGURE 6). This alllorejection can be effectively prevented by blocking the T-cell chemokine receptors CCR5 and CXCR3 with TAK-779 or the binding of CD154/CD40L to CD40 primarily expressed on activated T cells with anti-CD154/CD40L blocking antibody (FIGURE 6) (22, 114).

In the B6 (H-2^b^) mouse ACE transplanted with a couple of dozen islet equivalents from MHC-mismatched DBA/2 (H-2^d^) mice, these intracameral alloislets are progressively rejected although they are exposed to an immune-privileged environment (22). The allorejection starts at 14 days posttransplantation and ends up with the disappearance of all intracameral islet grafts at ∼50 days posttransplantation. Noninvasive, longitudinal, intravital microscopy visualizes that many activated effector T cells expressing green fluorescent protein (GFP) infiltrate into the ACE where 70% of them attack and destroy islet cells in intracameral allografts. GFP-labeled T cells have intimate contact with apoptotic islet cells. These T cells contain lysotracker-labeled lytic granules and infiltrate into allogeneic islets in parallel with islet destruction. Intracameral alloislets in recipients rendered diabetic by streptozotocin (STZ) injection undergo a quick failure of antihyperglycemic action and the actual T cells significantly increase their travel speed when infiltrating into alloislets (22). TAK-779 effectively slows down the initial infiltration of T cells into intracameral alloislets, dynamics of intracameral alloislet-infiltrating T cells, and decreases the relative proportion of the ruffled T cells in intracameral alloislets, thereby delaying allorejection (FIGURE 6). These activated effector T cells constitute a main culprit for intracameral alloislet rejection but one that is slower than that observed under the kidney capsule (22).

It is most likely that T cells infiltrate into the ACE and intracameral islet grafts through abundantly fenestrated endothelia lining capillaries in revascularized grafts. These endothelia share characteristics with those in in situ pancreatic islets, but not with those, which is nonfenestrated, in the recipient iris (FIGURE 6) (29, 46, 124, 132). Furthermore, revascularized vessels can appear in islets transplanted into the ACE as early as 3 days posttransplantation and reach quite a high density within one month after grafting (4). The abundant capillaries with well-fenestrated endothelia appear to be the major reason why allogeneic islets are rejected in the immune-privileged ACE (FIGURE 6) (29). In addition to the leakage of intragraft capillaries, the breakdown of blood-ocular barrier induced by ocular stress and inflammation resulting from transplantation surgical procedures are also likely to be involved (FIGURE 6) (115, 116). Furthermore, the neogenesis of lymphatic vessels in eyes bearing islet grafts may contribute to ocular immune privilege disruption. In fact, lymphatic vessel endothelial hyaluronan receptor 1 appears in these eyes but not in intact contralateral eyes in syngeneic NOD recipients suffering from autoinflammation (21). These findings reveal that a relatively small amount of intracameral allogeneic islets can induce disruption of the blood-ocular barrier that allows T cells to migrate into the ACE to destroy intracameral allogeneic islets in a CD4+ and CD8+ cell-dependent manner. These effects can be counteracted by alleviating T-cell-mediated inflammation with TAK-779 (FIGURE 6). They also substantiate that the ACE technology can serve as the most advantageous approach to in vivo cellular imaging of transplant rejection in a serial and noninvasive manner (22). This approach will benefit clinical islet transplantation that is almost exclusively allotransplantation (256).

The alloislet rejection is well recapitulated in the ACE and under the kidney capsule of recipient mice rendered diabetic by STZ injection (114). In C57BL/6 recipients, intracameral islets and those under kidney capsules, isolated from DBA/2 mice, undergo full rejection within 90 days and 50 days, respectively, with a corresponding loss of antihyperglycemic ability. This demonstrates that recipient mice reject alloislets inserted into the ACE more slowly than those under the kidney capsule (114). Intriguingly, 70% of alloislets in the ACE and 50% of them under the kidney capsule survive longer than 400 and 300 days, respectively, after five treatments with anti-CD154/CD40L blocking antibody at 3 and 1 day(s) before transplantation, the day of transplantation and 3 and 7 days after transplantation without further immunosuppression. This immune intervention effectively induces allotolerance (FIGURE 6) (114). In tolerant mice whose ACE was initially engrafted with alloislets, 70% alloislets subsequently transplanted under the kidney capsule survive ∼400 days without immunosuppression. By contrast, only 30% of the alloislets subsequently transplanted under the kidney capsule survive in the tolerant mice with the initial transplantation of alloislets under their kidney capsule (114). Furthermore, AH levels of several cytokines in the alloislet-bearing ACE of tolerant recipient mice significantly differ from those of recipient mice that are rejecting alloislets. There are elevations in the cytokines IL-4 and TGF-β2 as well as IL-5, but reductions in the Th1/Th17 cytokines IL-1β, IL-17α, and IFN-γ in the former recipient mice in comparison with the latter (114). Moreover, the recipient baboon whose ACE is engrafted with baboon alloislets shows reduced DTH (114). Such alterations in cytokine profiles and DTH responses support that tolerance to alloislets after transient treatment with anti-CD154/CD40L blocking antibody involves induction of Th2 cells and suppression of Th1/Th17 cells as well as both intracameral and systemic immune regulation. This together with the finding that alloislets subsequently transplanted under the kidney capsule survive longer in recipient mice whose ACE is initially engrafted with alloislets, compared to mice only receiving alloislets under their kidney capsule, suggests that ACAID induction takes part in the alloislet transplant tolerance.

Intracameral allografts act as a heavy burden on ocular immune privilege and undergo rejection when they are vascularized. However, this should not be only seen as a pitfall. On one hand, this situation can be made use of to study allorejection in a unique way as discussed above. On the other hand, intracameral allorejection can be intervened by local immunosuppression that produces little or no systemic side effects.

## 4. ACE TECHNOLOGY

**FIGURE 7.**
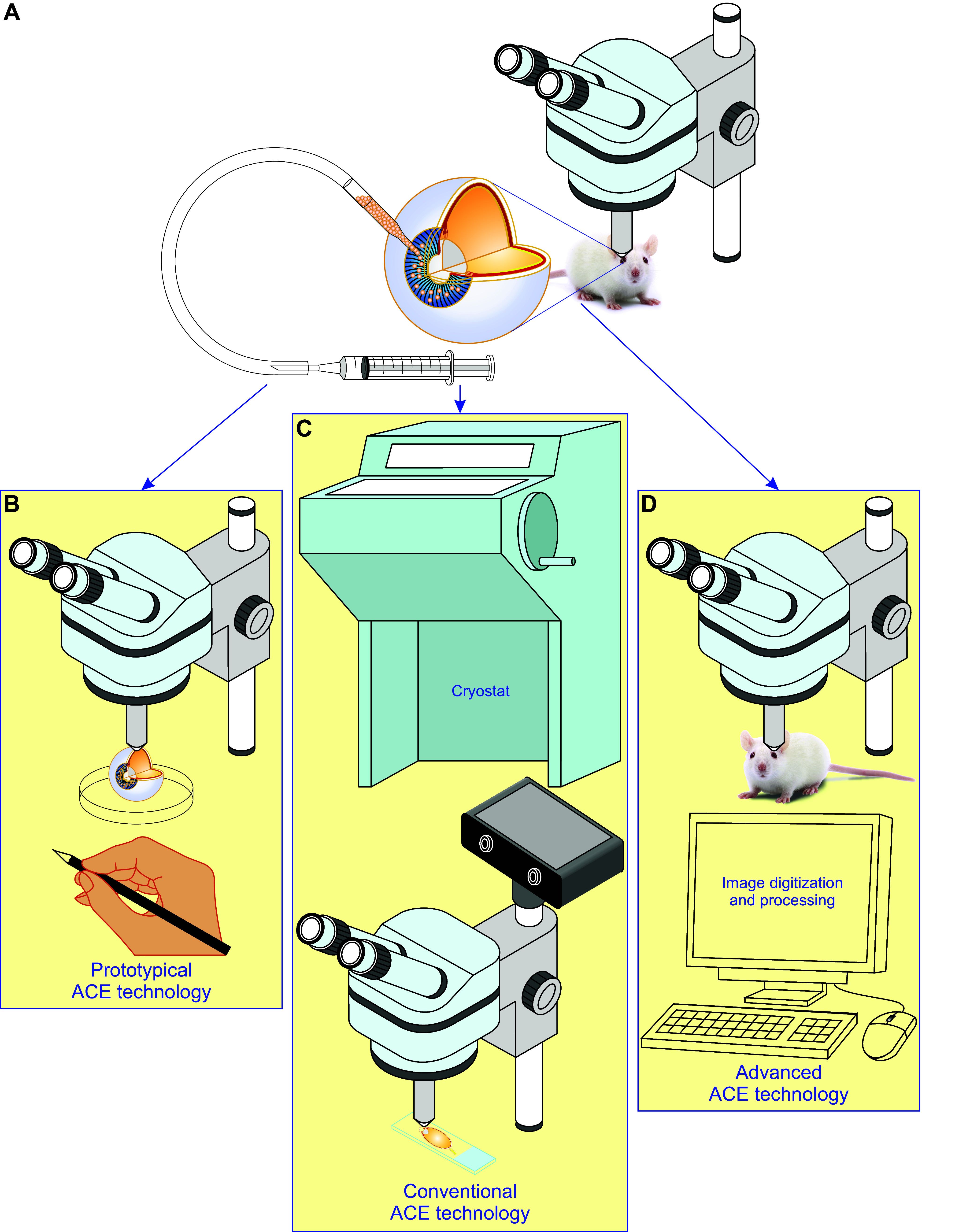
Evolution of the anterior chamber of the eye (ACE) technology. This technology is based on anatomical, optical, and immunological features of the ACE and has been developed with the advance of microscopic tools, fluorescent labeling, and image digitization/processing as well as relevant knowledge. *A:* ACE technology is built up essentially from transplantation of a batch of cells, pieces of tissues and organs, artificial biomaterials, pharmaceuticals, or abiotic substances into the ACE and has evolved into the following three versions. *B:* prototypical ACE technology, established by van Dooremaal in 1873, simply entails insertion of grafts into the ACE followed by crude observation of intracameral grafts and documentation with freehand drawing (3). *C:* conventional ACE technology is formed by combining ACE transplantation, a series of in vitro assessments typically by classical histology and microscopy, and objective data analysis and has been widely employed in different fields of biomedicines (38). *D:* advanced ACE technology is upgraded as a versatile tool with its own uniqueness by advanced microscopy, fluorescent labeling, and image digitization/processing on top of intracameral graft insertion, thus turning noninvasive, longitudinal, and intravital microimaging of different cells, tissues, and organs into reality (4–6).

**FIGURE 8.**
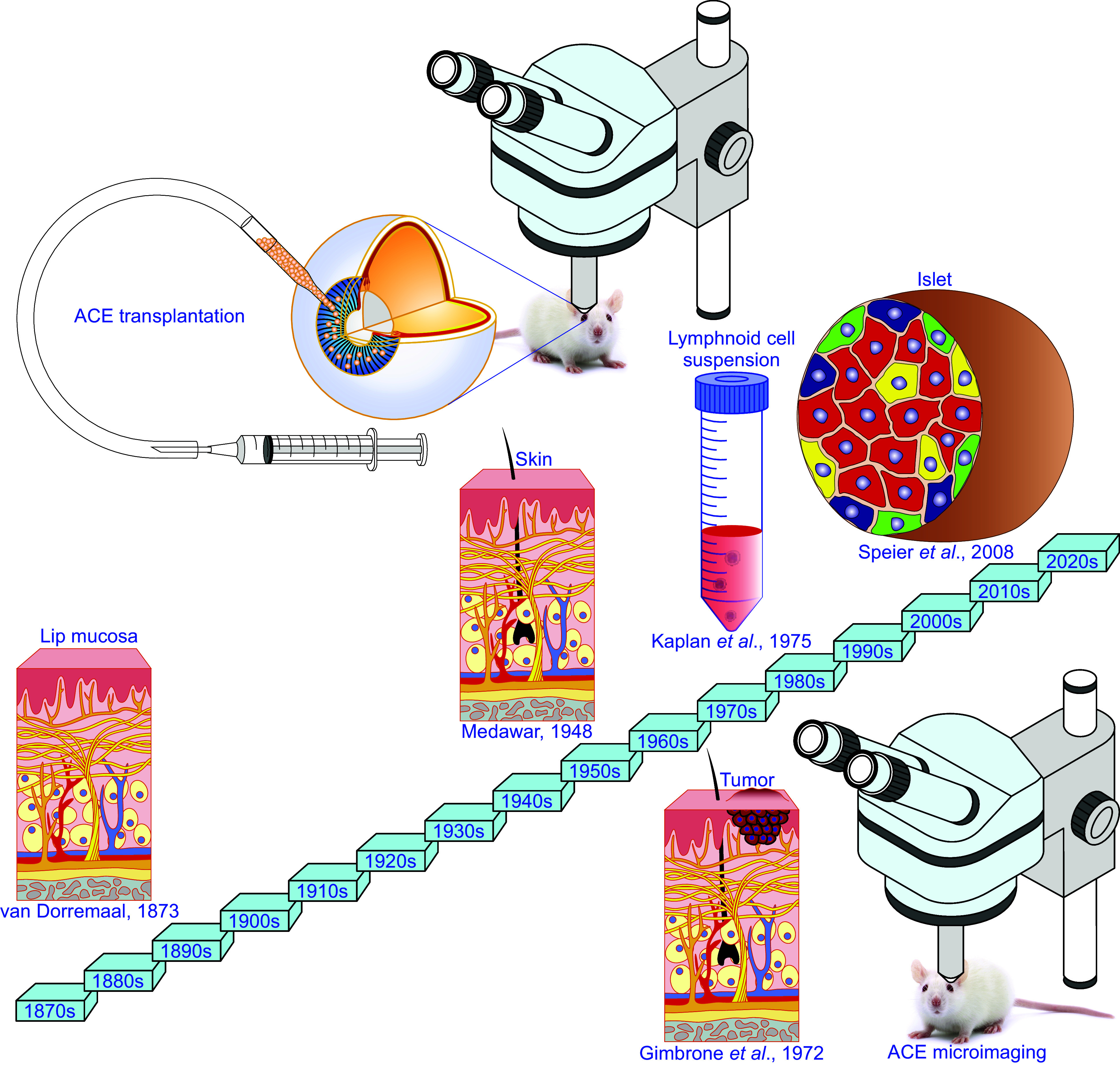
Major achievements by applying the anterior chamber of the eye (ACE) technology. Since its birth, the ACE technology has been successfully applied in various studies on different cells, tissues, and organs as well as artificial biomaterials, pharmaceuticals, and abiotic substances. These studies have brought the following major achievements. In 1873, van Dorremaal (3) found prolonged survival and progressive growth of homologous lip mucosa in the ACE of dogs and rabbits. This finding lays a solid foundation for the development of the concept of ocular immune privilege (3). Seventy-five years later, Medawar (38) immunologically dissected the prolonged survival of intracameral skin allografts of rabbits and coined the concept of ocular immune privilege. In 1972, Gimbrone et al. (372) found that intracameral tumor grafts stopped growing and entered “dormancy” if they were not vascularized and revealed the importance of antineovascular therapy against tumors. Soon after that, Kaplan et al. (149) inoculated allogeneic lymphonoid cells into the rat ACE and proposed “lymphocyte-induced immune deviation” that was subsequently corrected into ACAID. In 2008, Speier et al. (4, 5) succeeded in microimaging intracameral islets in a noninvasive, longitudinal, and intravital manner.

Although the anatomical, optical, and immunological features of the ACE naturally exist as a solid foundation for the ACE technology, they were not recognized until 1873 (3). In that year, van Dooremaal made the first successful transplantation of living tissues into the ACE of mammals and unintentionally created the prototypical ACE technology (FIGURES 7 AND 8) (3). It is so named because by using it, he was only able to do crude characterization of intracameral transplants due to the lack of appropriate research tools and relevant knowledge at that time. In fact, his data were just presented in nine freehand drawings even without a piece of photograph in the first publication on transplantation into the ACE known to history.

Soon after that, the approach to grafting cells, tissues, and organs into the ACE has widely been combined with different in vitro techniques and in particular classical histology, optical microscopy, and electron microscopy. Such a combination has substantially upgraded the prototypical ACE technology, implicitly being accepted as the conventional ACE technology and broadly adopted into different fields of biomedicines (FIGURES 5 AND 7). However, the conventional ACE technology was still more or less the same as the prototypical ACE technology for in vivo applications. With them, the gross morphology of intracameral grafts is seen through the transparent cornea directly and clearly by using the naked eye, a magnifying glass, or a classical microscope (6). The optical advantage of the ACE has so far been utilized minimally in in vivo studies. Both the prototypical and conventional ACE technology do not reach the level where the structure, function, and dynamics of intracameral grafts can be noninvasively, longitudinally, and intravitally microimaged. This technological gap has prevailed because of the limited functionality and in particular poor resolution of optical tools and the absence of advanced fluorescent labeling methods (6).

By the early 2000s, the popularization of advanced microscopy like confocal and multiphoton fluorescence microscopy and genetically encoded fluorescent indicators created necessary and sufficient conditions for noninvasive, longitudinal, and intravital microimaging of intracameral grafts. We caught the opportunity to bridge the aforementioned technological gap and developed advanced ACE technology (FIGURES 5, 7, AND 8) (4, 5). It turned noninvasive, longitudinal, and intravital microimaging of intracameral grafts into reality. This resulted in the unique intravital microimaging platform enabling high-resolution, noninvasive longitudinal visualization of the structure, function, and viability of intracameral grafts, exemplified by pancreatic islets, in a 3-D mode (4, 5). Since then, we and others have widely used the advanced ACE technology in different fields enabling conceptual research advances (4–7, 15–37, 61, 62). The methodological aspects of the two modules of the ACE technology, i.e., transplantation into the ACE and intravital microimaging of intracameral grafts, using the cornea as a natural body window, as well as the pros and cons of the ACE technology, are discussed below.

### 4.1. Transplantation into the ACE

In the history of medicine, transplantation has been developed to replace diseased or damaged body parts (257–262). Unlike clinical applications, transplantation of grafts into the ACE was originally established for research into the etiology of cataract formation rather than for restoration of diseased or damaged cells, tissues, and organs in the body (3). Transplantation into the ACE is simple without either cutting or suturing (4–6, 15, 59, 62). It has its own great power in research and holds great potential for therapeutic substitution for some diseased, damaged, or devitalized microorgans, like pancreatic islets, parathyroid glands, and other endocrine tissues. Satisfactory transplantation of grafts into the ACE requires quite a few considerations such as the preservation and quality assessment of grafts as well as strain or genetic pairings between transplant donors and recipients just as in the case of other types of transplantations (3, 4, 6, 26, 36, 38, 57, 256, 263–269). These considerations are beyond the scope of the present review article and there is therefore no need for further discussions. Some points critical and specific for ACE transplantation are discussed below.

#### 4.1.1. Graft size and number for ACE transplantation.

In principle, grafts for transplantation into the ACE should be sized to minimize the corneal hole for graft insertion and maximize their survival. The actual grafts have very tiny blood vessels, which are unable to be anastomosed to those in the host ACE (FIGURE 5). Indeed, intracameral grafts can undergo satisfactory vascularization, but it takes about 1 month if they are placed on the iris of recipients. Therefore, these grafts can neither receive oxygen, nutrients, and growth/survival factors from, nor eliminate metabolic wastes properly to the blood of recipients, thus suffering from warm ischemia before their vascularization (6). Some intracameral grafts without contact with the iris can hardly be vascularized during the whole period of transplantation. Intracameral grafts critically rely on the diffusion of the AH for their survival in the early posttransplantation period or even during the entire posttransplantation period. In fact, during the period from the time when grafts are extracted from donors to the time when sufficient vascularization occurs in intracameral grafts, cell damage and death are inevitable, especially in their cores. This happens because the necessary supplies from the AH cannot effectively diffuse into and harmful metabolites cannot properly be removed from the core of grafts. Apparently, the size of grafts has a positive correlation with the severity of these problems, the bigger the worse. Reducing the size of grafts can yet be regarded as a good measure. Pancreatic islets less than 250 μm in diameter can relatively easily be prepared and satisfactorily survive without appreciable cell death in their cores. Based on this, other preparations, like adrenals, ovaries and pituitary, cardiac and skeletal muscles, and the brain and spinal cord, should be cut into small blocks with diameters less than 250-μm or 250-μm-thick slices. The size of cell aggregates and engineered organoids can be controlled during production and should be less than 250 μm in diameter (7). For example, we have designed and generated surrogate islets from human induced pluripotent stem cells (hiPSC-islets) with less than a 250-μm diameter, which are readily placed into the ACE (FIGURE 5) (7). Noteworthy is that the preparation of other types of naturally existing tissues/organs in appropriate sizes is not easy. They are large, attached to unwanted tissues, and have to be dissected out, chopped, and trimmed into pieces in a suitable size (6, 34).

How many grafts should be placed into one ACE depends on the purpose of the designed transplantation and the volume of the ACE of recipients. In the case of islet transplantation for glycemic control, a maximum of 500 islets and a total of 38,000 islet equivalents (IEQs) can fit into one ACE of mice and monkeys, respectively (26, 28). If intravital microimaging is needed, theoretically, just one graft with satisfactory engraftment in one ACE can do the job. However, in practice, several to a couple dozen islets are inserted into one ACE to compare different grafts in the same ACE and to gain the best experimental sample size with the least number of experimental animals. Furthermore, several to a couple of dozens of islets are engrafted separately from each other on the iris. These separately engrafted islets stay free of mechanical squeezing, physical interaction, and opaque cover. Therefore, they keep their optical visibility, microarchitecture, function, and viability optimal to satisfy the conditions for high-resolution intravital microimaging (6, 7). To evaluate the ability of grafts and in particular endocrine grafts, as best exemplified by pancreatic islets, to restore or improve the functionality of dysfunctional organs in situ, the amount of grafts containing the largest possible mass of functional cells should be transplanted into the ACE. Noteworthy is that such a large amount of grafts squeeze each other and eventually aggregate together in the ACE. The aggregated grafts significantly change their morphology without clear boundaries. They are not suitable for intravital microimaging at high resolution but nevertheless survive and function well (26, 36).

#### 4.1.2. Genetic labeling of grafts before ACE transplantation.

Before ACE transplantation, there is usually a need to genetically label appropriately prepared grafts with fluorescent protein biomarkers or biosensors for in vivo microimaging of cellular and molecular events of interest (FIGURE 5) (6, 24, 25, 36). Indeed, there are various genetically modified mice in which different fluorescent protein biomarkers or biosensors are specifically expressed in certain types of tissues or cells. The corresponding cells, tissues, or organs isolated from these mice can be directly transplanted into the ACE without being labeled with any fluorescent protein biomarkers or biosensors. However, quite a few types of donor cells, tissues/organs especially isolated from humans need to be transduced with genetic vectors encoding fluorescent protein biomarkers or biosensors of interest (4, 5, 24, 25, 36). This step is done for in vivo microscopic characterization of intracameral grafts at molecular and cellular levels. If there is no need to perform intravital microimaging of intracameral grafts, this step is not applicable.

#### 4.1.3. ACE transplantation modes.

**FIGURE 9.**
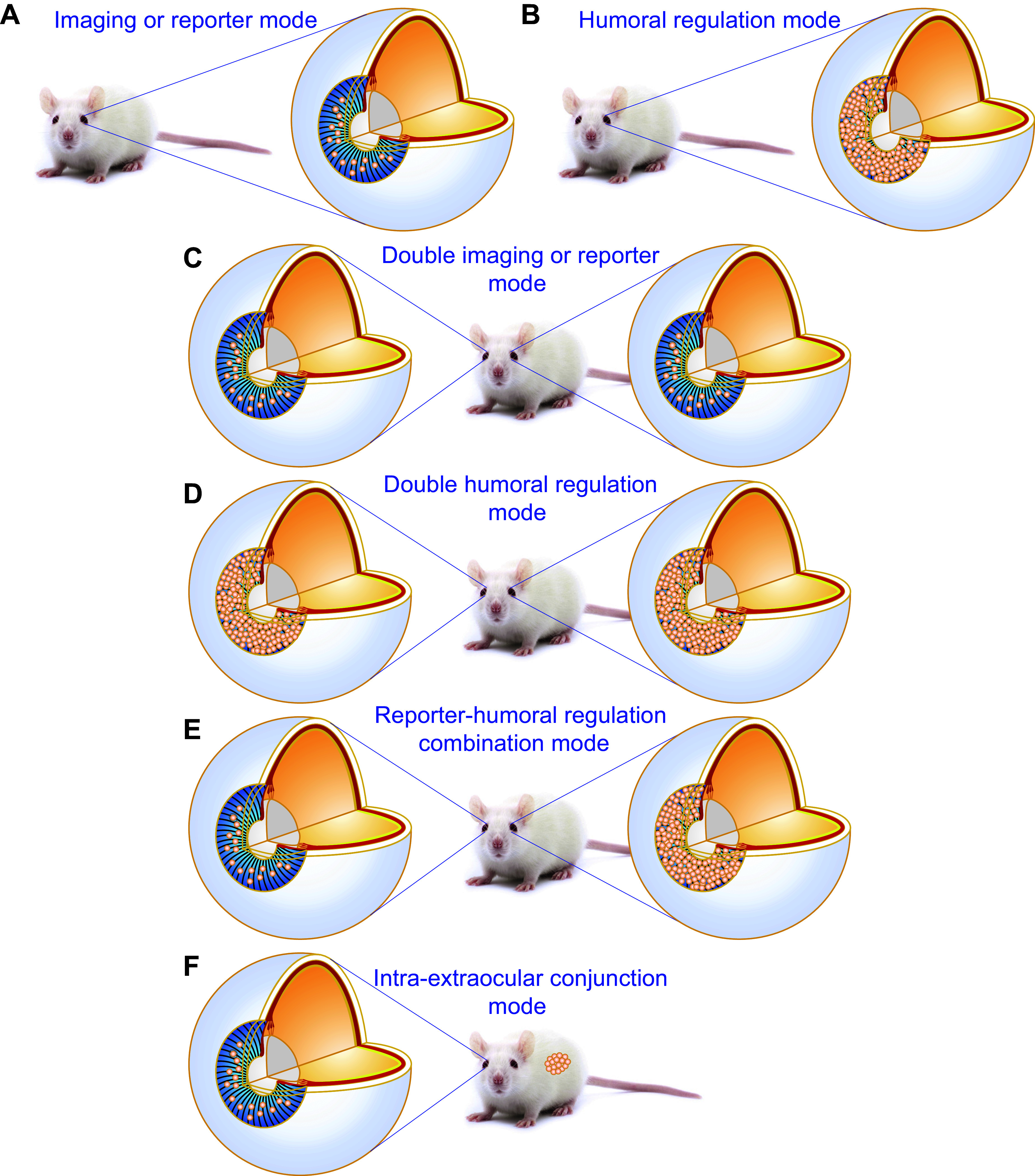
Modes of the anterior chamber of the eye (ACE) technology. The technology can work in the following six different modes. *A:* imaging or reporter mode. It is best qualified for noninvasive, longitudinal, intravital microscopy of intraocular grafts. *B:* humoral regulation mode. It is suitable for evaluation of the humoral action of biomolecules produced by a large enough number of intracameral grafts. *C:* double imaging or reporter mode. In addition to the merits of the single mode, this double mode is optimal for paired experiments and helps obtain a larger sample size from the same number of recipients. *D:* double humoral regulation mode. It has the same merits as mentioned in *C*. In addition, it is the only way to increase the number of intracameral grafts to produce enough humoral factors for humoral regulation like normalization of hyperglycemia. *E:* reporter-humoral regulation combination mode. It serves the purpose of microimage intracameral grafts and observes their roles in humoral regulation in parallel in the same recipients. *F:* intra-extraocular conjunction mode. On one hand, it has the same merits as either imaging or reporter or humoral regulation mode. On the other hand, it can deal with the large number or size of grafts that cannot fit into the ACE.

According to the number and layout of grafts placed into the ACE, there are two basic modes. One mode is termed “imaging or reporter mode” (FIGURE 9). In this mode, several to a couple of dozens of islets are inserted into the ACE. These intracameral grafts are separated from each other at the beginning and gradually engraft without obvious aggregation (4–7, 63). These nonaggregated grafts do not suffer mechanical squeezing, physical interaction, and opaque cover, display optimal optical visibility, and preserve normal microarchitecture, function, and viability as in their orthotopic sites (4–6). Therefore, their morphology, function, and viability can be microimaged noninvasively, longitudinally, intravitally, and at single-cell resolution. Moreover, these variables also alter in phase with those of their counterparts in situ (4–6). Hence, the intracameral grafts serve as faithful reporters of their counterparts in situ (4–6). The other basic mode is referred to as “humoral regulation mode” (FIGURE 9). This mode aims to make use of the full capacity of the ACE for holding the maximal number of intracameral grafts that can produce enough corresponding humoral factors for humoral regulation in the recipient’s body (4, 6, 23, 28–30, 84, 95). These intracameral islets gather in the ACE and even squeeze each other forming a clump in this limited space. In such an islet clump, individual islets are markedly distorted and cannot be distinguished from each other, thus being inappropriate for microimaging. However, they can survive and function even better than in situ pancreatic islets. Therefore, the ACE can bear enough islets to keep glucose homeostasis under tight control in recipients although it has a limited space compared to other sites (4, 23, 28–30, 84, 95). In fact, 50 syngeneic islet equivalents engrafted on the iris of C57BL/6 (B6) mice, rendered diabetic by injection of STZ, have sufficient antihyperglycemic capacity to mitigate recipient survival (95). Nevertheless, at least 125–150 syngeneic islet equivalents are required to normalize hyperglycemia (29, 95). Indeed, the intracameral islets function as an extraordinary controller of glucose homeostasis (4, 23, 28–30, 84, 95).

In the body, other transplantation sites are unpaired. By contrast, two ACEs are paired in the same recipient. Based on this, in addition to the two basic modes, different modes of islet transplantation into the ACE can be arranged to fulfill different research aims and objectives. Three additional ones have been derived (FIGURE 9). Transplantation of islets into the ACE on each side of the same recipient at the same time or at a certain interval forms “double microimaging or reporter mode,” “double glycocontrol or metabolic mode,” the latter also being called “double humoral regulation mode” in a broader term (FIGURE 9). These modes bring several benefits. They offer an excellent opportunity to implement optimally paired experiments. Both ACEs of the same recipient can be transplanted with genetically modified islets and wild-type-control ones, respectively, or with the same number of islets similar in size from the same donor followed by different local treatments. This can reduce the number of animals used in experiments, increase the space for more grafts, and importantly make experiments have high-quality controls. The “double glycocontrol or metabolic mode” appears to be the only choice if one ACE transplanted with a maximum number of islets still cannot meet experimental requirements such as normalization of hyperglycemia. In addition to these two double modes, “humoral regulation-imaging combination mode” or simply “combination mode” can be established in the same recipients (FIGURE 9). The combination mode allows microimaging of islet cytoarchitecture, function, and viability and also the regulation of glucose homeostasis in the same recipients simultaneously. If used together with other islet transplantation sites, such as the hepatic portal system, renal subcapsules, intra-abdominal cavity, omental pouch, gastrointestinal wall, subcutaneous tissue, skeletal muscle, bone marrow, pancreas, spleen, lung, brain, testis, and thymus, one can formulate another interesting mode, namely, “intra-extraocular conjunction mode” (FIGURE 9). The other sites can accommodate significantly more grafts like islets than the ACE, whereas no one other than the ACE is optically accessible. By taking advantage of the superiority of extraocular islet transplantation sites in conjunction with the merits of the ACE, one can estimate what happens in extraocular islet grafts by imaging intraocular ones.

#### 4.1.4. Uncomplicated, least-invasive, and bloodless transplantation into the ACE.

Following the above preparations, the last step, i.e., insertion of high viable grafts in a good size into the ACE takes place (FIGURE 5) (6, 24, 25, 34, 36). It starts with a gentle aspiration of grafts into a transplant-delivering micropipette, whose back end is connected to a threaded plunger syringe through Tygon tubing. To minimize corneal damage and ease transplant-delivering micropipette insertion into the ACE, the tip of the transplant-delivering micropipette is beveled and lightly heat polished, and its final diameter typically ranges from 150 to 300 μm. In the case where the size of transplanted tissue/organ pieces has to be relatively larger, the tip diameter of the transplant-delivering micropipette can be enlarged appropriately but not too much since the large corneal hole can disturb ocular immune homeostasis and hardly be healed. As soon as transplants are aspirated into the micropipette, a tiny corneal hole is created by carefully puncturing the cornea of recipients with the tip of an insulin syringe needle (29 G). Usually, continuous inhalation of a mixture of 2.5% isoflurane and 40% oxygen is performed via a nose mask to anesthetize recipients. In this way, the onset, depth, duration, and termination of anesthesia are easily controllable. Immediately following the creation of the tiny corneal hole, the tip of the micropipette preloaded with grafts is cautiously inserted into the ACE and preloaded grafts are smoothly injected into the ACE. Of note, patience and caution are needed mostly when the glass micropipette is pulled out from the corneal hole. Prudent and slow pulling can avoid the escape of transplants from the hyperbaric ACE. In practice, transplantation of islets or other tissues/organs into the ACE can be done almost noninvasively without bleeding. In such a controlled and careful way, the ACE technology does not really give rise to any insults or infections in the eye. Nevertheless, caution should be exercised with ACE transplantation surgery. Poorly performed ACE transplantation can cause severe eye diseases like uveitis, although there has been no report on such problems after carrying out many cases of ACE transplantation in our and other laboratories.

### 4.2. Intravital Micro-imaging of Intracameral Grafts

Noninvasive, longitudinal, and intravital microimaging of the real-time morphology and function of intracameral grafts, also known as ACE microimaging, was established in 2008 (FIGURES 5 AND 7) (4, 5). With confocal/multiphoton laser scanning microscopy, we have, for the first time, done ACE microimaging of islets engrafted in the ACE of live rodent recipients (FIGURES 5 AND 7) (4, 5). Basically, the procedures established in our work are applicable for other grafts even without modification. They proceed as follows (4, 5). Anesthetizing a recipient animal is the first step, wherein it is critical to select appropriate anesthetic agents that do not influence parameter(s) of interest and the physiological status of recipients. In addition, a note of caution is worth articulating at this point regarding anesthetic selection for different experiments applying the ACE platform. Some anesthetics markedly interfere with the parameter(s) of interest and/or are fraught with obvious pitfalls that disturb or even prevent experiments from being implemented. For example, isoflurane induces impaired glucose-stimulated insulin secretion (GSIS) and hyperglycemia as well as irregular eyeball/iris movements (5, 270). Therefore, this anesthetic agent is only used for the purpose of imaging cytoarchitecture, light scattering, vascularization, and innervation of intraocular islets as a snapshot in addition to performing transplantation surgery because of its merits of being easily controlled. However, isoflurane is inappropriate for continuous, long-last recordings of events such as [Ca^2+^]_i_ responses to intravenous injection of high glucose in intracameral islets (4, 19). A combination of fluanisone, fentanyl, and midazolam has been validated to be a satisfactory selection for imaging [Ca^2+^]_i_ dynamics in intracameral islets following intravenously injected glucose (4, 19).

Next, the anesthetized recipient is stabilized with a head holder that angles the eyeball-bearing grafts to a proper orientation suitable for microimaging and placed under an upright microscope. The eyeball of the recipient is immobilized with an eyeball holder before microimaging. Then, if parameter(s) of interest cannot be imaged in intracameral grafts, fluorescence labeling has to be performed. [Ca^2+^] indicators, fluorescent dyes for detecting apoptosis, and fluorophore-conjugated antibodies to T cells have successfully been used in labeling intracameral islets (4, 5).

Eventually, grafts are microimaged under illumination with appropriate laser beams (FIGURES 5 AND 7) (4, 5). During microimaging, it is important to maintain an appropriate, but not too deep anesthetic depth, where the recipient is unresponsive to the uncomfortable fixation of its head and eyeball for high-resolution data acquisition, in addition to the physiological status including normal body temperature, respiratory, and hemodynamic stability. Image size and acquisition speed should be optimized to gain the best match of spatial versus temporal resolution with morphological versus functional data.

Comprehensive imaging software has been instrumental for the ACE technology (FIGURE 7). It turns high-speed digitization of images into reality resulting in fast acquisition of images with high spatiotemporal resolution and complex reconstruction of vivid dynamics of image signals from and 3-D structure of intracameral grafts. Importantly, it enables objective quantification and analysis of different parameters of intracameral grafts (4, 5).

### 4.3. Strengths and Weaknesses of the ACE Technology

**FIGURE 10.**
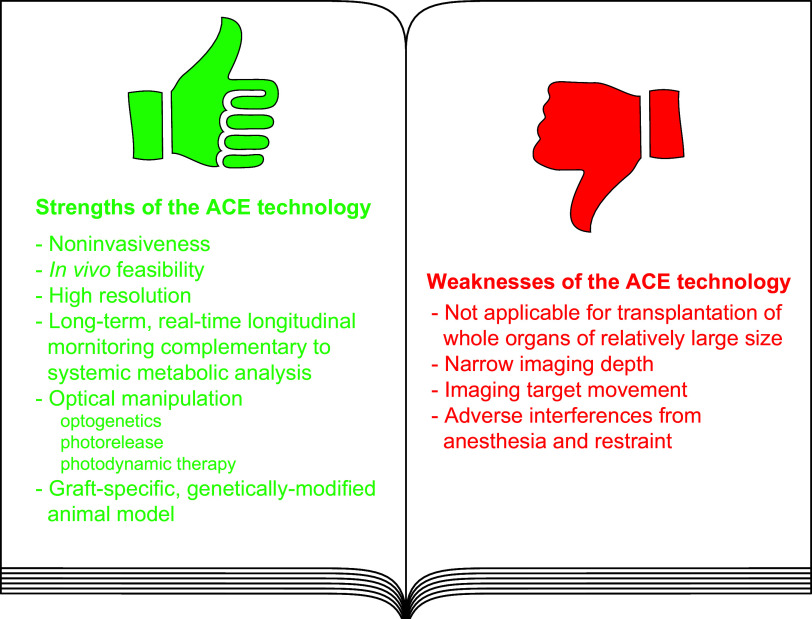
Strengths and weaknesses of the anterior chamber of the eye (ACE) technology. The major strengths and weaknesses of the ACE technology are summarized herein.

The ACE technology has pros and cons (FIGURE 10). Nevertheless, the pros outweigh the cons of this technology. Both are discussed in detail below.

#### 4.3.1. Strengths of the ACE technology.

The main strengths of the ACE technology are tied to its competence in microimaging. The ACE microimaging manifests a range of advantages over other imaging approaches such as invasive in vivo microscopy, in vitro microscopy, and conventional noninvasive in vivo imaging modalities (FIGURE 10) (5, 14, 15, 271).

First and foremost, the noninvasiveness of the ACE microimaging is most important. It breaks down a great barrier for application of in vivo microscopy (FIGURE 10) (14). As a matter of fact, application of conventional in vivo microscopy is strictly limited by two main factors invasiveness and its resultant effects like inflammation. This approach can only noninvasively access either tissues/organs on the body surface such as skin, oral mucosa, and some parts of the eye, or the digestive, respiratory, urinary, and reproductive tracts by being combined with endoscopy (272, 273). It is not possible to reach other tissues/organs like pancreatic islets, muscles, and the brain without surgical operation due to insurmountable anatomical hurdles. In addition, unavoidable surgical incisions and pressure squeeze for arrangement of microscope objectives and optical probes inevitably give rise to tissue damage, local inflammation, and even systemic traumatic stress. These adverse consequences disturb microscopical observations causing potential false interpretations of data. Apparently, invasive in vivo microscopy is not suited for longitudinal microimaging because of the long-term consequences of surgical damage and their derived ethical issues. These problems have been solved by the ACE technology, demonstrating that it can serve as an optimal habitat for ectopic cells, tissues, or organs, as exemplified by islets, which survive and behave the same way as their counterparts in situ and importantly are optically exposed. With the ACE technology, there is no longer any anatomical barrier or a need for surgical operation when intravitally microimaging optically inaccessible cells, tissues, or organs. The great advantage of the ACE technology is that it allows longitudinal, noninvasive in vivo microscopy of most, if not all, of the body’s cells, tissues, or organs without surgical damage and resultant inflammation (4–6, 15).

Next, the ACE technology has elevated in vitro microscopic research to in vivo levels and turned nondestructive in vivo microimaging of morphology, viability, and functionality of intracameral islets and other grafts into reality (FIGURE 10) (4, 5, 15–17, 20, 23, 27, 29, 30, 32, 84, 95, 274, 275). The morphological, survival, and functional profiles of cells, tissues, and organs visualized in the presence of in vivo cell-cell and/or cell-extracellular matrix interactions and interstitial milieu, as well as innervation and blood supply, reflect true physiology and/or pathology (4, 5, 15–27, 29–33, 60, 84, 95, 274–281). On the contrary, in vitro findings obtained with dispersed single cells and isolated tissues or organs in the absence of the in vivo milieu are not fully representative of the complex physiological and/or pathological situations in the living organism. Moreover, dispersed single cells and isolated tissues or organs also suffer from various stresses like chemical insults, enzymatic attacks, and mechanical disturbances during their preparation. These adverse effects definitely deteriorate the architecture, function, and viability of dispersed single cells and isolated tissues or organs. Obviously, one should cautiously extrapolate the in vitro findings to the in vivo situations (6, 14) and revisit the knowledge acquired in vitro by applying advanced in vivo technical means like the ACE technology.

In addition to the noninvasiveness and in vivo feasibility, the high-resolution feature is another strength of the ACE technology (FIGURE 10). This has upgraded the resolution of noninvasive intravital imaging of the body’s tissues and organs to submicrometer scales. Undeniably, before the development of the ACE imaging, one has established a variety of noninvasive in vivo imaging modalities including bioluminescence imaging, computer-assisted tomography (CT), elastography, magnetic particle imaging, magnetic resonance imaging (MRI), positron emission tomography (PET), photoacoustic imaging, and ultrasonography, which remarkably contribute to biomedical sciences and clinical practice (5, 14, 15, 271). However, they all underperform in terms of resolution in comparison to the ACE imaging (5, 14, 15, 271).

Another merit, i.e., proficiency in repetitively and longitudinally microimaging of the same specimen makes the ACE technology more elegant and versatile than invasive in vivo microscopy and in vitro microscopy (FIGURE 10) (5, 14, 15). Chronic diseases like diabetes proceed along the gradual progression of different pathogenic processes, many of which can be reversed at their earlier stages (282). Undoubtedly, understanding the temporal profile of these processes and identifying when each of these processes reaches the point of no return, e.g., β-cell demise in the development of diabetes, are of paramount importance.

The ACE technology can also be used as a complementary system deciphering pathological mechanisms of disease and in particular diabetes by simultaneously measuring multiple optical signals that reflect the architecture, function, and viability of intracameral grafts like islets and systemic metabolic parameters, e.g., blood glucose, insulin, and C-peptide levels (FIGURE 10) (283).

The optical features of the eye endow the ACE technology with an exclusive advantage that makes it a noninvasive in vivo optical manipulation site. Intracameral transplants are ideally accessible to light flashing used in optogenetics, photorelease, and photodynamic therapy without any invasive operation (FIGURE 10) (284–286). This exclusive advantage brings the idea of noninvasive handling of the cellular function and viability of intracameral grafts into reality through different ways, e.g., optogenetic stimulation of heterologously expressed light-sensing proteins, photo-uncaging of light-sensitive caged compounds and photoactivation of photosensitizers (284–286). In addition, experimental evidence shows that the pupillary light reflex appears to serve as the “innate optogenetic module” operating in reinnervated intracameral islets that release insulin under the tight control of light exposure (30).

Transplantation of tissues or organs from animals carrying modified genes throughout their body into the ACE can create a graft-specific, genetically modified recipient model, which allows investigations of the role of the gene of interest only in the graft but not in any part of the recipient’s body (FIGURE 10). We have transplanted Ca_V_β_3_ knockout mouse islets into the ACE of recipient wild-type mice rendered diabetic by high-fat diet feeding and STZ injection. The obtained data show that knockout of the Ca_V_β_3_ gene in islets produces a stronger antidiabetic role than wild-type islets. This excludes the possibility that the enhanced GSIS and blood glucose clearance observed in general Ca_V_β_3_ knockout mice results from other tissues and organs like the autonomic nervous system rather than islets (32).

#### 4.3.2. Weaknesses of the ACE technology.

The ACE technology also has certain weaknesses (FIGURE 10). The application of the ACE technology to awaken free-moving animals is unable as of yet. Data acquired from anesthetized and immobilized animals may bear potential pitfalls since anesthesia and physical restrain can influence imaged grafts through the recipient’s neural and humoral systems (270).

The ACE does not allow transplantation of sizable tissue blocks or whole organs as replacement therapy due to its limited space and lack of specific microenvironments required for the performance of certain grafts, e.g., hematopoietic stem cells and progenitor cells (FIGURE 10).

The movements of intracameral grafts resulting from the respiration, heartbeat, pupil constriction, and dilation of recipients prevent the resolution capacity of confocal/multiphoton microscopy in the ACE technology from fully playing out (FIGURE 10). Therefore, one needs to minimize any risk to cause movements of intracameral grafts. Adequate immobilization of the head and eyeball, appropriate adjustments of the level of anesthesia, and optical or pharmacological control of pupil constriction and dilation are effective measures. By doing so, the ACE technology can reach a crude subcellular resolution that is able to distinguish between the cytoplasm and nucleus. So far, the image depth of the ACE technology is limited (FIGURE 10). The use of longer wavelength lights for excitation is a way to improve the maximum imaging depth.

## 5. MAJOR ACHIEVEMENTS GAINED USING THE ACE TECHNOLOGY

With the help of the ACE technology, as a versatile biomedical research platform with its own uniqueness, a wide range of basic knowledge and in-depth understanding of a variety of cells, tissues, and organs as well as artificial biomaterials, pharmaceuticals and abiotic substances inserted in the ACE have been obtained (3–9, 15–17, 20, 23, 27–30, 32, 36, 38, 48, 57, 63, 84, 95, 102–104, 274, 275, 280, 281, 287–371). Unfortunately, given space limitations, we can only discuss major achievements gained using the ACE technology below (FIGURE 8).

### 5.1. The Invention of the ACE Technology

van Dooremaal inserted various foreign objects and different living issues/cells, such as tiny pieces of paper, cork, shot, hair, conjunctiva, lip mucosa, skin, periosteum, and human epidermis, into the ACE of mice, rabbits, or dogs to find the causes of cataract formation (3). Unfortunately, he failed to reach his original aim but fortunately witnessed intriguing phenomena in a new era of transplantology (FIGURE 8). As expected, the intracameral foreign objects as irritants triggered acute inflammation being subjected to an extrusion process and eventually causing panophthalmitis although they were encapsulated in occasional cases (373, 374). However, the living tissues were well integrated and engrafted in the ACE. As matter of a fact, two findings were unexpected in this seminal work. One is a considerable prolongation in the survival of mouse skin grafts placed into the ACE of dogs. The other is the apparent formation of “tumor-like” structures from intraocularly allotransplanted mucous membranes of the lip (3). This not only verifies that both homologous and heterologous grafts are more accepted in the ACE compared to conventional transplantation sites but importantly leaves a solid basis for establishing the concept of the ocular immune privilege (FIGURE 8) (3, 38).

### 5.2. Important Findings Obtained with the ACE Technology

During the progression and popularization of the ACE technology, researchers in different fields satisfactorily adopted this technology into their research. Among them, Medawar, Streilein, and Folkman revisited the ACE with great success (38–40, 48, 57, 102–104, 372, 375).

Medawar and colleagues (38–40) have carefully characterized homologous skin grafts from outbred rabbits transplanted into the ACE in contrast to conventional body sites from the perspective of immunology (FIGURE 8). He witnessed that homologous skin grafts survived for prolonged times when inserted into the ACE in the absence of intragraft vascularization. On the contrary, these grafts were promptly rejected if placed at conventional body sites and into the ACE in the presence of intragraft vascularization. Furthermore, the survival of these intracameral skin homografts is independent of blood supply because vascular stagnation is not causally linked to the breakdown of these grafts. These findings significantly contribute to the establishment of the concept of the ocular immune privilege (38–40).

In the late 1970s, the Streilein group (45, 98, 150, 152–157, 159, 160) set out to inoculate a broad range of antigens, including viruses, haptenated cells, soluble proteins tumor antigens, and histocompatibility antigens into the ACE (FIGURE 8). They revealed that these inoculated antigens escaped from the ACE although they did not bring about appreciable ocular angiogenesis. In fact, these inoculated antigens can be carried by intracameral APCs into the bloodstream through the ocular trabecular meshwork and then enter the systemic immune apparatuses thymus and spleen. Consequently, these inoculated antigens actively induced the ACE-dependent, antigen-specific immune deviation, i.e., ACAID (FIGURE 8) (41, 44–46, 48, 57, 98, 99, 102, 112, 148–150). This helped us to deeply and comprehensively understand the ocular immune privilege and adds another layer of complexity to this specific immunological concept (see sect. 3.2).

Folkman and coworkers (372) have realized tumor biology as another area of opportunity for the ACE technology. They suspended small, viable, and mitotically active tumors in the AH of the rabbit ACE where they survived but maintained avascular and were reluctant to grow for 6 weeks. However, the same tumor inserted into the iris not only underwent satisfactory vascularization but also exponential growth within 2 weeks. These findings made them establish the concept of tumor dormancy and point out the importance of antineovascularization in the treatment of tumors (372). Furthermore, they tried to find out the possible reason for tumor dormancy by placing tumors at various distances from the iris vessels. They believe that intracameral tumor dormancy results from a lack of blood supply rather than cell cycle arrest or immune control because dormant tumors start growing when moved closer to the iris (375). This is open to discussion because vascularized and nonvascularized tumors in the ACE are exposed to the systemic immune environment and immune-privileged niches, respectively, besides different blood supplies. Furthermore, owing to the above, the ACE appears to be suitable for understanding the influence of inflammation on the progression of vascularized tumors, but not nonvascularized tumors.

The ACE technology has also been used to nondestructively and intravitally microimage pancreatic neuroendocrine tumors (376). The data show that preoncogenic islets from the Rip1Tag2 transgenic mice were well engrafted and vascularized on the iris and spontaneously developed into β-cell tumors when grafted into the ACE of syngeneic recipients. Interestingly, a majority of differently intracameral preoncogenic islet grafts underwent oncogenic growth, whereas a small portion of them maintained their original size. These intracameral tumors shared hallmarks with those occurring in the pancreas in situ. Furthermore, treatment with the angiogenesis inhibitor sunitinib counteracted intracameral tumor angiogenesis and growth. This work demonstrates the suitability of the ACE technology for cancer research as well as anticancer drug screening (376).

### 5.3. Comprehensive Refinements of the ACE Technology and Resulting Knowledge

The prototypical ACE technology has been combined with different biomedical approaches into the conventional ACE technology soon after its birth and further refined into the advanced ACE technology along with the development of the ACE-based microimaging (FIGURE 8) (4–6). The ACE imaging based on advanced microscopy and image digitization as the central module not only allows high-resolution imaging but also objective image quantification and documentation of intracameral transplants, among which islets transplanted into the ACE are the first and best examples (FIGURE 8) (4–6). A variety of cells, tissues, and organs have indeed been characterized with the conventional ACE technology, but the obtained information is limited and even biased (3, 8, 9, 38–40, 48, 57, 102, 287–369). They have yet to be further investigated by using the advanced ACE technology to be comprehensively understood. By using the advanced ACE technology, we have documented how intracameral grafts undergo dynamic engraftment and vascularization and preserve their specific functions as exemplified by pancreatic islets grafted into the ACE. As a representative of intracameral grafts, islets are well adapted to the ACE of recipients, thus comfortably settling down and intimately integrating into this new habitat (4–7, 15–17, 20, 23, 27–30, 32, 36, 63, 84, 95, 274, 275, 280, 281, 370, 371). The knowledge acquired, by using the advanced ACE technology, on engraftment and vascularization, innervation, glycemic control, [Ca^2+^]_i_ dynamics, β-cell mass, and insulin secretory capacity of intracameral islets is further detailed below.

#### 5.3.1. Engraftment, graft vascularization, and survival.

High-resolution in vivo microscopy shows that islets mostly settle down on the iris and infrequently adhere to the cornea shortly after being placed into the ACE. The islet grafts and host iris or cornea are intimately attached to each other with a contact interface but not intertwined (4–6). Before being vascularized, intracameral islets can be nourished by AH and comforted by ocular immune privilege. Subsequently, those islets engrafted on the iris become vascularized by iridic blood vessels and progressively integrate with iridic tissue. Within about a month, they reach full vascularization and engraftment (4–6).

Engrafted islets can have a contact interface of different sizes with the recipient iris. The ones with small contact areas are spherical, while the others with large contact areas are somewhat flat in shape. Nevertheless, both engraft and function equally well. As stated earlier, fully engrafted islets can be arranged into different modes (FIGURE 9). Importantly, the cytoarchitecture of intracameral islets resembles that in in situ pancreatic islets (4–6).

Longitudinal real-time imaging witnesses that intracameral islets grafted on the iris begin their vascularization at day 3 posttransplantation. At this point, they just recruit some functional blood vessels in their peripheral area in direct contact with the iris (4). Afterward, these vessels progressively extend throughout an islet graft building up primary microvascular networks 2 weeks after transplantation. Thereafter, intraislet microvascular networks become denser. Their density reaches a plateau at week four posttransplantation. Then, intraislet microvascular networks remodel their capillaries, which become tortuous and uniform in diameter and interwoven with each other. However, the diameter of intraislet vessels decreases twice from day 3 to 14 posttransplantation, becomes one red blood cell wide, and remains unchanged thereafter (4). This reflects that newly formed intraislet vessels develop into mature capillaries (4).

In vascularized islet grafts, most or even all of the endothelial cells come from host iridic vascular networks. In cases where freshly isolated islets carry their own endothelial cells, some of them remain to join iridic endothelial cells forming mosaic vasculatures (29, 377, 378). Whereas freshly isolated islets have their own vascular endothelial cells and those experiencing cultivation have none, both undergo the same degree of revascularization over the posttransplantation period, thanks to the dominant contribution of host endothelial cells. However, freshly isolated islets were revascularized at a higher rate than cultured ones. Unexpectedly, the former takes longer to reverse diabetes than the latter when transplanted into the ACE of mice with STZ-induced diabetes (29). It is likely that donor islet’s own endothelial cells make a minor contribution to early islet revascularization, which however does not significantly elevate the vascular density or ameliorate the function of islet grafts. Transmission electron microscopy verifies that the ultrastructure of donor and host endothelial cell-lining blood vessels in intracameral islet grafts is normal and resembles that of in situ pancreatic islets. There is no discriminative ultrastructural feature between donor and host endothelial cells. Thin endothelial cell bodies with fenestrations covered by a diaphragm form intraislet capillary walls where a thin basement membrane slightly separate vascular endothelial cells from islet cells (29). Of note, vascular endothelial cells become fenestrated although they stem from the host iridic blood vessels that are formed by nonfenestrated endothelial cells (46, 124, 132).

As is well known, islets residing in the pancreas are highly vascularized (379, 380). Their vascular network is critical for maintaining the physiological extracellular milieu in the islet by driving effective exchange between blood plasma and interstitial fluid (379). This is because the intraislet vascular network is responsible for indispensable housekeeping, like nutrient supply to and metabolic waste disposal from islets, and also for well-timed transportation of hormonal factors, such as insulin, glucagon, and incretins to and from islet cells (381). Hence, the intraislet vascular network plays an important part in the preservation of the structural integrity and functional competence of islets and in the orchestration of systemic metabolism and glucose homeostasis (379, 381, 382). In addition, interactions between vascular endothelial cells and islet cells are important for the optimal function of islets (382). All of the above emphasize that vascularization of islet grafts in the ACE or for that matter at any site in the host body is important. Nevertheless, intracameral grafts, which are floating in the AH and free of vascularization can in rare cases manage to sustain their viability due to the partial replacement of blood supplies by AH circulation.

It is noteworthy that the aforementioned findings were obtained in syngeneic rodent models. There are evident differences in engraftment and vascularization between intracameral mouse and human islet grafts. A slower engraftment and vascularization to a lesser degree occurs in the latter (16). Intracameral islet grafts enjoy long-term survival in autorecipients and syngeneic recipients and can survive from transplantation until euthanasia or natural death (4, 27). Vascularized alloislets can be fully rejected 50 days after transplantation into the ACE (22).

#### 5.3.2. Innervation.

Intracameral islets adequately restore their sympathetic and parasympathetic innervations, which are similar to those in in situ pancreatic islets (30). In intracameral islets engrafted on syngeneic mouse iris, innervation is likely to follow in parallel with vascularization during posttransplantation. Sparse autonomic nerve endings grow to the surrounding area of the islet grafts on day 3 after transplantation. Sympathetic terminals enter the islet grafts, mostly associated with blood vessels, at day 15 posttransplantation and undergo outgrowth over the period from day 15 to day 30 posttransplantation. Subsequently, sympathetic endings expand to the adjacent area of islet cells and raise their density and complexity to a plateau at day 90 posttransplantation. Parasympathetic innervation similarly proceeds in the islet grafts. Parasympathetic terminals grow close to intraislet vessels in the beginning and arrive in close vicinity to islet cells. Although similar, parasympathetic innervation goes somewhat slower than sympathetic innervation. By taking advantage of transgenic mice whose cholinergic neurons in the parasympathetic nervous system specifically express GFP in their somas and processes, in vivo microimaging of parasympathetic innervation of intracameral islets has also been conducted. It shows that the distribution of GFP-positive cholinergic neurons and processes in innervated wild-type intracameral islets does not differ from those in in situ endogenous pancreatic islets (30).

Iridic autonomic outputs mediated by the two classical neurotransmitters acetylcholine and noradrenaline act as important regulators of hormone secretion from intracameral islet grafts (30). Therefore, one can harness the pupillary light reflex to noninvasively manipulate hormone secretion from the innervated intracameral islets (30). In fact, 300 mouse islets grafted into the ACE of mice rendered diabetic by STZ injection are able to normalize hyperglycemia. Interestingly, increased circulating insulin and glucagon and reduced blood glucose levels occur following ambient light exposure. Plasma insulin levels decrease and blood glucose concentration increases in recipient mice carrying intracameral islets when shifted from bright ambient light to darkness and vice versa when the recipient mice are moved from darkness to light. These phenomena do not happen in recipient mice transplanted with islets under the kidney capsule. The effects induced by light exposure mainly result from the activation of muscarinic receptors of intracameral islet cells by acetylcholine released from iridic parasympathetic terminals. Furthermore, glucose tolerance tests show that the ambient light enhances blood glucose clearance in mice with intracameral islets but not grafted with islets under the kidney capsule. The enhancement is effectively prevented by the muscarinic receptor antagonist atropine and recapitulated by the muscarinic receptor agonist pilocarpine (30). Evidently, the iridic parasympathetic nerve branches out into intracameral islet grafts to modulate their endocrine function (30).

Especially worthwhile is that there are some interstrain differences in parasympathetic innervation of islets between B6 and 129X1 mice, the latter islets are less innervated by cholinergic terminals than the former ones. Interestingly, these interstrain differences remain in intracameral islet grafts (30). Recipient mice whose ACE is grafted with B6 mouse islets show clear light-induced facilitation of blood glucose clearance, whereas recipient mice carrying 129X1 mouse islet grafts in the ACE do not. This pinpoints both the importance of islet parasympathetic innervation in the regulation of GSIS as well as interstrain differences (30). Furthermore, obvious interspecies differences in autonomic innervation exist between human and mouse islets. Unlike intracameral mouse islets, intracameral human islets are innervated by a few parasympathetic endings and some sympathetic terminals, the latter preferentially innervating vascular smooth muscle cells and seldom contacting endocrine cells. These autonomic terminals innervate intracameral human islets in the same way as they do in the human pancreas in situ. Owing to their relative scarcity, these autonomic terminals are not likely to influence insulin secretion following ambient light exposure (30).

#### 5.3.3. Glycemic control.

The antihyperglycemic efficacy of intracameral islets has been investigated (4, 6, 23, 25, 28–30, 84, 95). Initially, the obtained data show that 300 mouse islets transplanted into the ACE of mice with STZ-induced diabetes can fully reverse hyperglycemia in recipient mice and keep them nondiabetic until surgical excision of the islet graft-bearing eye (4). These intracameral islets also satisfactorily rectify impaired glucose tolerance in STZ-induced diabetic mice, making these recipient mice regulate blood glucose as effectively as control mice (4). Two independent observations show that 125 and 150 syngeneic islets grafted into the ACE of mice rendered diabetic by STZ injection are enough to achieve normoglycemia (29, 95). According to these two observations, ∼125–150 mouse islets engrafted in the ACE of syngeneic recipients are believed to be the marginal mouse islet mass for glycemic normalization (29, 95). By contrast, in a human donor islet-nude mouse recipient model, 1,000 intracameral human islet IEQs (500 human IEQs in each ACE) have been verified to sufficiently reverse STZ-induced hyperglycemia (26). In the case of the allogeneic nonhuman primate model, initial transplantation of 20,000 IEQs, and subsequent addition of 18,000 IEQs on day 292 posttransplantation appreciably diminish fluctuations in fasting blood glucose (28).

Taken together with the data obtained from rodents, the nonhuman primate model substantiates that the ACE, regardless of species, can serve as a suitable transplantation site where islet grafts not only survive but also optimally release insulin to effectively regulate glucose homeostasis (4, 28–30, 84, 95). Importantly, the ACE can significantly optimize islet graft survival and function in comparison to other transplantation sites. Therefore, intracameral islet grafts more effectively control glucose homeostasis than those at other transplantation sites (4, 28–30, 84, 95). Interestingly, the hypoglycemic effect of 50 intracameral mouse islets is equivalent to that produced by ≥200 renal subcapsular mouse islets (95).

#### 5.3.4. [Ca^2+^]_i_ dynamics.

[Ca^2+^]_i_ dynamics in islets is critical for various functions of β-cells and have been thoroughly studied in dispersed single islet cells and isolated islets under nonphysiological in vitro conditions (383–388). Under such conditions, observed [Ca^2+^]_i_ dynamics and corresponding signaling pathways may not authentically reflect those operating in vivo. Obviously, in vivo [Ca^2+^]_i_ dynamics in islet cells under different physiological and pathophysiological conditions inevitably need to be explored.

[Ca^2+^]_i_ dynamics in islets engrafted in the ACE has carefully been characterized by using noninvasive, longitudinal, and intravital microscopy at the early stage of the development of the advanced ACE technology (4, 5). Intracameral islet grafts labeled with the Ca^2+^ indicators Fluo-4 and Fura-Red show a marked [Ca^2+^]_i_ increase in response to intravenous injection of the sulfonylurea compound glibenclamide, which brings about the sequential events from adenosine triphosphate-sensitive K^+^ (K_ATP_) channel closing to [Ca^2+^]_i_ elevation through plasma membrane depolarization, voltage-gated Ca^2+^ (Ca_V_) channel opening and Ca^2+^ influx in β-cells (4, 383, 385, 386, 388–390). This [Ca^2+^]_i_ response quickly occurs 30–40 seconds after injection of glibenclamide and is typically manifested as a rapid initial rise and a subsequent sustained increase. Intriguingly, individual β-cells in the intracameral islet graft simultaneously elevate their [Ca^2+^]_i_ with a similar pattern following glibenclamide injection. The synchronization of [Ca^2+^]_i_ response is attributed to the tight coupling between individual β-cells of the intracameral islet graft. In addition, the glibenclamide-induced [Ca^2+^]_i_ response in the same islet cells of the intracameral islet graft can be monitored longitudinally (4).

Later on, by combining ACE imaging and a transgenic mouse model with the β-cell-specific expression of the Ca^2+^ sensor protein GCaMP3, glucose-induced [Ca^2+^]_i_ dynamics have been carefully characterized (19). In overnight-fasted mice, β-cell [Ca^2+^]_i_ in the intracameral islet stays relatively stable but quickly increases to its maximal level after glucose injection and thereafter gradually drops down to a lower plateau superimposed with or without oscillations (19). Furthermore, these in vivo [Ca^2+^]_i_ responses are more quickly evoked by glucose challenges than in vitro [Ca^2+^]_i_ responses in isolated islets. The former exhibits the initial [Ca^2+^]_i_ peak in less than 1 minute after glucose injection, whereas the latter does so when glucose stimulation lasts for 3–4 minutes (19, 391, 392). Hence, there is an obvious difference between in vivo and in vitro β-cell [Ca^2+^]_i_ dynamics. Unquestionably, the advanced ACE technology and in particular ACE imaging is a powerful tool to understand in vivo islet physiology as here demonstrated by β-cell [Ca^2+^]_i_ dynamics (4, 19).

Recently, the Rutter group (370, 371) has made two interesting observations on in vivo [Ca^2+^]_i_ dynamics in islets by taking advantage of the advanced ACE technology. They demonstrated that highly coordinated [Ca^2+^]_i_ dynamics as a functional signature of islet β-cell connectivity operates in murine and human islets grafted into the ACE as well as in zebrafish islets in situ. This [Ca^2+^]_i_ dynamics originates from temporally and transcripromically defined leader β-cells or alternatively “hub” cells and spreads out in waves over neighboring follower β-cells. In this context, these leader β-cells not only serve as originators but also pacemakers or coordinators under control of blood glucose concentration (370). In vivo [Ca^2+^]_i_ dynamics in intracameral islet grafts have been used to report β-cell connectivity and functionality in in situ islets of recipient mice fed high-fat/high sucrose diet following bariatric surgery (371). Bariatric surgery is known to ameliorate diabetes by mitigating insulin resistance and β-cell deterioration. However, nondestructively, longitudinally, and intravitally microimaging the progressive recovery process of deteriorated β-cells and relevant mechanistic events has not been technically doable before the establishment of the advanced ACE technology. ACE microimaging of [Ca^2+^]_i_ dynamics in intracameral islet grafts as reporters of islets in situ shows that vertical sleeve gastrectomy enhances β-cell [Ca^2+^]_i_ dynamics and increases the number and strength of connections between β-cells in association with increased circulating GLP-1 levels. These findings indicate that vertical sleeve gastrectomy gives rise to elevated incretin production and thereby drives the progressive recovery of β-cell functionality reflected by restoration of highly coordinated [Ca^2+^]_i_ dynamics (371). These two observations convincingly illustrate the uniqueness of the advanced ACE technology in noninvasive, longitudinal, and intravital microimaging.

#### 5.3.5. **β**-Cell mass and insulin secretory capacity.

Given the importance of functional β-cell mass in the maintenance of glucose homeostasis and in the development of diabetes, there is an ultimate need to nondestructively follow the in vivo dynamics of β-cell mass and function (393–398). The advanced ACE technology and in particular ACE imaging has successfully enabled the characterization of in vivo dynamics of β-cell mass and insulin secretory capacity of intracameral islet grafts (6, 18).

β-cells contain abundant zinc-insulin crystals within insulin secretory granules. This endows these cells with unique light scattering properties that allow nondestructive, longitudinal in vivo imaging of β-cell mass and insulin secretory capacity of intracameral islet grafts without fluorescence or genetic labeling (6, 18). Quantification of the light scattering signal with ACE imaging can reliably determine in vivo dynamics of both intraocular islet volumes and insulin granule density, which reflect real-time β-cell mass and insulin secretory capacity, respectively, under different metabolic conditions, such as fed and/or fasting states. This methodology is superior to other methods due to noninvasive, longitudinal, intravital, and high-resolution data acquisition with few or no artificial influences (6, 18, 399–405).

Indeed, light scattering signals emitted from intracameral islets increase and decrease following acute exposure to low and high glucose for a sufficient period of time. For example, overnight fasting can increase light-scattering signals in islets due to the substantial accumulation of insulin-secretory granules. The volume and density of light scattering signals can indeed be used to estimate insulin secretory capability, but are not proportionally related to insulin secretory capability. These signals also originate from other cellular components in intraocular islets in addition to zinc-insulin crystal-containing secretory granules. Furthermore, the total zinc-insulin crystal-containing secretory granules exceptionally outnumber the release-competent ones in GSIS. As is well known, a β-cell contains a total of ∼10,000 insulin secretory granules, but only 50 of them are docked and primed in the immediately releasable pool for the initial phase of GSIS (406, 407).

## 6. CONCLUSIONS AND FUTURE DIRECTIONS

The understanding of human health and disease critically relies on how much detail available technologies can resolve especially in the living body without invasive manipulation. The establishment and application of the ACE technology have turned out to be a significant step in the right direction. The ACE technology has resolved a wide range of physiological enigmas and medical dilemmas by combining intracameral insertion of cells, tissues, and organs as well as reagents with ACE microimaging (4–7, 15–37, 61, 62). There are still a number of unanswered questions, and there is much room for improvements and optimization for the present state-of-the-art ACE technology. Many interesting aspects in biomedicine and in particular drug discovery and disease pathogenesis as well as treatment await advances by employing the ACE technology.

The oxygen/nutrient-rich and immune-privileged ACE performs as the special nursery bed where transplanted cells, tissues, and organs are optimally engrafted, richly vascularized, and densely innervated (4–6, 30, 90–93). Therefore, their morphological characteristics, cellular composition, and organotypic specificity are preserved. Intracameral grafts demonstrate satisfactory survival and perform physiological functions and are sometimes even better than when being in their in situ location, as exemplified by pancreatic islet grafts (4, 6, 28–30, 84, 95). These merits lead to a broadening of the use of the ACE technology to most, if not all, body parts including skin, nervous tissue, muscles, endocrine organs, kidney, liver, lymphoid organs, and even amniotic membrane, placenta, fertilized eggs, and somatic cell- or stem cell-derived organoids and tumors (3, 7–9, 22, 34, 38–40, 58, 62, 63, 287–369, 376). However, only some of them like intracameral islets, thymus, amniotic membrane, and kidney grafts have so far been satisfactorily microimaged in a nondestructive, longitudinal, and intravital manner with regard to their structure, function, viability, and development under physiological conditions (4–7, 34, 61–63). Among them, intracameral islet grafts are best studied using the advanced ACE technology (4–6). Substantial knowledge has been obtained with regard to in vivo dynamics of islet graft vascularization, [Ca^2+^]_i_ responses to systemically administrated glucose and functional β mass as well as on the development of pancreatic buds and hiPSC-islets applying noninvasive, longitudinal, and real-time microimaging, the core modality of the advanced ACE technology (4–7, 15, 18, 19, 21–33, 60, 63, 84, 276–279). Other intracameral grafts have yet to be studied in this way.

The ability to noninvasively, intravitally microimage intracameral transplants has greatly upgraded the ACE technology but also added requirements for stability when imaging grafts. Therefore, the ACE technology is at the moment only applicable to restrained animals under general anesthesia (6). Such physical and pharmacological management inevitably bring about various unwanted influences when imaging grafts. It is challenging to apply this technology in awake, free-moving animals. Collective expertise in advanced electronics, nanotechnology, and microscopy is expected to bring solutions to these challenges by developing intracamerally microimaging probes for transplantation. Even in restrained and anesthetized animals, it is difficult to circumvent problems related to target movements during ACE microimaging. This significantly compromises the spatiotemporal resolution of the microimaging modality. Currently, this problem prevents the ACE technology from resolving fine subcellular organelles such as mitochondria and insulin-secretory granules. A specific confocal/multiphoton microscope equipped with a high-performance, continuous autofocus function in combination with a servomechanism-driven target-chasing system will overcome this limitation in resolution.

The limited imaging depth and working distance are further issues negatively affecting the ACE technology. In this perspective, the ACE technology underperforms compared to conventional noninvasive in vivo imaging modalities like CT, MRI, and PET and leaves room for improvement (271).

Humanized mouse models have been demonstrated to be useful in modeling human diseases and appraising new drugs and treatment regimens in human cells, tissue, and organ grafts (408–411). The ACE technology has satisfactorily been used in immunodeficient mice, but not yet in mice humanized with the human immune system. The latter are superior to the former in answering human-specific and clinically relevant questions. The powerful combination of ACE technology and advanced humanized mouse models will create further breakthroughs in biomedical fields focused on human health and disease.

Neurons, myocytes, and endocrine cells are electrically excitable and perform their specific missions, like information transmission, muscle contraction, and hormone secretion, under tight control of complex electrical events resulting from the concerted activities of a variety of ion channels (412). Understanding how these ion channels operate in vivo under physiological and pathological circumstances is important for clarifying their role in electrically excitable cells in health and disease. Indeed, electroencephalography, electroneurography, electromyography, and electrocardiography work effectively in appraising electrical activities in some of these cells. However, very little is known about the in vivo behavior of ion channels in these cells because they are normally localized in areas where patch clamp analysis can hardly be done. Most, if not all, of our knowledge on ion channels in electrically excitable cells has been obtained with in vitro preparations that lack functional nerve endings, vascular networks, and in vivo interstitial fluid as well as suffer from different types of harmful stress during isolation and cannot fully preserve in vivo gained phenotypes (6). Apparently, what is observed in these in vitro preparations will not reliably reflect what happens in vivo and thereby unavoidably introduce biases and errors. There is no doubt that the intravital characterization of ion channels in healthy and diseased neurons, myocytes, and endocrine cells is necessary and important. By creating reasonable accessibility to these electrically excitable cells engrafted on the iris, the ACE technology offers the great possibility for intravital characterization of ion channels by combining the patch-clamp technique and microimaging approaches.

The 3-D organoid technology shows promising potential to advance regenerative medicine and arouses great attention. The dedicated efforts have resulted in the satisfactory construction of various organoids derived from somatic or stem cells (413, 414). Importantly, bioengineering organoids from human stem cells and in particular iPSCs cannot only produce an unlimited amount of human organoids but also immune rejection-free human iPSC-derived organoids from the patient’s own somatic cells, potentially removing the need for scarce donors and immunosuppressive regimens (413–415). They are expected to be able to curatively and safely treat a damaged or diseased body part in patients with relevant diseases such as Parkinson’s disease, myocardial infarction, and diabetes (413, 414). To this end, they must be capable of undergoing vascularization, engraftment, remodeling, and maturation to fulfill their specific functions. Poor vascularization cannot appropriately link bioengineered organoid grafts to the circulatory system of recipients resulting in insufficient survival and engraftment. Unsuccessful integration with recipient tissues at the transplantation site may prevent bioengineered organoid grafts from being fully functional. In addition, organoids derived from human stem cells including iPSCs can hardly reach their full maturity in vitro and are likely to do so in vivo (7, 63). However, they may contain undesirable cell populations causing tumorigenic risks and in vivo maturation is difficult to investigate at conventional transplantation sites (7, 63, 416–422). Apparently, all these barriers to clinical applications of bioengineered organoids are relatively long-term in vivo processes and need to be tackled with appropriate approaches. The ACE technology appears to be tailor-made for these tasks. It is highly qualified for microwitnessing long-term in vivo vascularization, engraftment, remodeling, and maturation as well as the safety of single miniature organoids noninvasively, longitudinally, and repeatedly at single-cell resolution. It qualifies as a potent tool for prescreening clinically appropriate organoids.

Indeed, intracameral islets well serve as drug targets to directly report pharmacological actions of systemic administrated drugs such as the antidiabetic agent liraglutide (26). Interestingly, the ACE as a confined space renders intraocularly applied drugs subjected to local pharmacokinetics, pharmacodynamics, and toxicology. In this context, the immunosuppressant rapamycin loaded in sustained-release microparticles placed into the ACE effectively prevented the rejection of intracameral allogeneic islet grafts (140). Local treatment of intracameral hiPSC-islets with intravitreally infused NNC55-0396 effectively promotes glucose-activated [Ca^2+^]_i_ dynamics in these islets (63). Hence, the ACE technology is expected to successfully contribute to in vivo pharmacological research.

The ACE technology integrated with suitable animal model systems and different approaches gives feasibility in modeling disease pathogenesis in vivo even with human cells, tissues, and organs in accordance with high ethical standards. The pathogeneses of certain diseases, e.g., diabetes, have been recapitulated and visualized by grafting pancreatic islets isolated from healthy and diseased donors including humans into the ACE of immunodeficient animals. Remarkably, the advanced ACE technology has broadened our horizons and helped us gain an in-depth understanding of the development of both type 1 diabetes and type 2 diabetes (18, 19, 23–25, 36, 423). It has succeeded in real-time, high-resolution tracking of the progression of type 1 diabetes by modeling autoimmune insulitis in the ACE where immune cell behavior and dynamics of functional β-cell mass have been well recapitulated (18, 423). Noninvasive, longitudinal, and intravital microimaging reveals the in vivo dynamic profiles of β-cell [Ca^2+^]_i_, function and mass as well as insulin resistance in intracameral islet grafts during the development of type 2 diabetes (19, 24, 25). The ACE technology has demonstrated the detrimental effects of excessively expressed β-cell Cav3.1 channels on GSIS and glucose homeostasis and the critical role of intraislet vascular aging in intracameral islet senescence (23, 36). Apparently, the advanced ACE technology can be adapted to visualize in vivo cellular and molecular pathogenesis occurring in other cells, tissues, and organs to enable an understanding of the true role of pathogenic events in different cells, tissues, and organs during disease development.

It should be emphasized that the human ACE is likely to host enough islets to normalize hyperglycemia in patients with diabetes although its narrow space accommodates fewer islets than other sites. This is because islets show more effective antihyperglycemic action when grafted in the ACE than in other sites (4, 6, 23, 25, 28–30, 84, 95). This may be true also for other endocrine tissues such as the thyroid, parathyroid, pituitary, adrenal, and sexual glands. It will be interesting to explore the possibility of making use of the ACE as a clinical transplantation site for different endocrine glands to treat human endocrine disorders. In fact, the preclinical application of the ACE technology in nonhuman primates has demonstrated the practicality of the technology in the curative treatment of diabetes (27, 28). Both baboon and macaque islets are well engrafted on the allogenic and autologous iris where they maintain normal cytoarchitecture and undergo full innervation and vascularization (27, 28). Transplantation of 38,000 baboon IEQs into an allogeneic baboon ACE rendered diabetic by STZ injection can ameliorate hyperglycemia without major adverse effects on eye function and structure. These preclinical studies support the concept that the human ACE can be safely used as a clinical islet transplantation site for treating diabetes (28). We are in the process of conducting clinical trials in patients with type 1 diabetes both in Europe and in the United States. Successful transplantation into the ACE has been carried out in one patient. We are establishing the protocols for insulin-dependent type 2 diabetes patients suffering from hypoglycemia unawareness. Outcomes of these clinical trials are anticipated to prevail over those observed with the hepatic portal system, the conventional site for clinical islet transplantation. From our studies in rodents and nonhuman primates, we are convinced that islets transplanted to the ACE will control glucose homeostasis in diabetic humans.

Furthermore, the proof of concept that Ca_V_β3-deficient islets show a great antidiabetic action suggests that genetic manipulation of human islets or their β-cells can significantly reduce the number of human islets needed for transplantation into the ACE (32). Clinical islet transplantation into the ACE may trigger and propel clinical transplantations of other organs like pituitary and parathyroid glands to treat dwarfism and hypoparathyroidism.

Numerous prospective avenues for ACE technology in laboratory research and clinical practice have yet to be delineated. The ACE technology may thus not only contribute to an in-depth understanding of the fundamentals of biomedical science but also to the prevention and cure of diseases like diabetes.

## GRANTS

This work was supported by grants from ERC-2018-AdG 834860 EYELETS, the Erling Persson Foundation, the Family Knut and Alice Wallenberg Foundation, Funds at Karolinska Institutet, Jonas and Christina af Jochnick Foundation, Strategic Research Program in Diabetes at Karolinska Institutet, Swedish Alzheimer Association, the Swedish Diabetes Association, Swedish Foundation for Strategic Research, the Swedish Research Council, and the Novo Nordisk Foundation.

## DISCLOSURES

S.-N. Yang is a consultant to Biocrine AB. P.-O. Berggren is the cofounder and CEO of BioCrine AB.

## AUTHOR CONTRIBUTIONS

S.-N.Y. and Y.S. prepared figures; S.-N.Y. and Y.S. drafted manuscript; S.-N.Y. and P.-O.B. edited and revised manuscript; S.-N.Y. and P.-O.B. approved final version of manuscript.
